# Honokiol Ameliorates Post-Myocardial Infarction Heart Failure Through Ucp3-Mediated Reactive Oxygen Species Inhibition

**DOI:** 10.3389/fphar.2022.811682

**Published:** 2022-02-21

**Authors:** Jianyu Liu, Minghai Tang, Tao Li, Zhengying Su, Zejiang Zhu, Caixia Dou, Yan Liu, Heying Pei, Jianhong Yang, Haoyu Ye, Lijuan Chen

**Affiliations:** ^1^ State Key Laboratory of Biotherapy and Cancer Center, National Clinical Research Center for Geriatrics, West China Hospital of Sichuan University, Chengdu, China; ^2^ West China-Washington Mitochondria and Metabolism Center, Department of Anesthesiology, Laboratory of Anesthesiology and Translational Neuroscience Center, West China Hospital of Sichuan University, Chengdu, China

**Keywords:** heart failure, myocardial infarction, honokiol, reactive oxygen species, UCP3

## Abstract

Post-myocardial infarction heart failure (post-MI HF) is one of the leading global causes of death, and current prevention and treatment methods still cannot avoid the increasing incidence. Honokiol (HK) has previously been reported to improve myocardial ischemia/reperfusion injury and reverse myocardial hypertrophy by activating Sirt1 and Sirt3. We suspect that HK may also have a therapeutic effect on post-MI HF. In this study, we aimed to investigate the efficacy and mechanism of HK in the treatment of post-MI HF. We found that HK inhibited myocardial reactive oxygen species (ROS) production, reduced myocardial fibrosis, and improved cardiac function in mice after MI. HK also reduced the abnormality of mitochondrial membrane potential (MMP) and apoptosis of cardiomyocytes caused by peroxide in neonatal cardiomyocytes. RNAseq results revealed that HK restored the transcriptome changes to a certain extent and significantly enhanced the expression of mitochondrial inner membrane uncoupling protein isoform 3 (Ucp3), a protein that inhibits the production of mitochondrial ROS, protects cardiomyocytes, and relieves heart failure after myocardial infarction (MI). In cardiomyocytes with impaired Ucp3 expression, HK cannot protect against the damage caused by peroxide. More importantly, in Ucp3 knockout mice, HK did not change the increase in the ROS level and cardiac function damage after MI. Taken together, our results suggest that HK can increase the expression of the cardioprotective protein Ucp3 and maintain MMP, thereby inhibiting the production of ROS after MI and ameliorating heart failure.

## Introduction

Heart failure (HF) is one of the leading global causes of death, with about 64.3 million patients currently living with HF worldwide (Shah et al., 2017; Disease et al., 2018). Nearly 70% of all HF syndromes can be attributed to underlying ischemic heart disease, which plays a pivotal role in the development and progression of HF with reduced and preserved ejection fraction (EF) (Elgendy et al., 2019). The data from cohorts enrolled in HF treatment trials suggest that almost two-thirds of HF cases are associated with obstructive coronary artery disease (CAD) (Abraham et al., 2002; Bristow et al., 2004; Taylor et al., 2004; Bardy et al., 2005; Cleland et al., 2005; Elgendy et al., 2019; Heallen et al., 2019). Among CAD, myocardial infarction (MI) is the most common cause of HF (Jenca et al., 2021).

MI is pathologically defined as cardiomyocyte death caused by an ischemic injury, which is the consequence of an imbalance in oxygen supply and demand (Thygesen et al., 2018). MI can cause cardiac systolic dysfunction, diastolic dysfunction, metabolic disorders, and perturbations of ionic balance (Kübler and Spieckermann, 1970; Pouleur and Hayashida, 1994; Jennings, 2013). It is estimated that a typical MI results in the loss of approximately one billion functional cardiomyocytes. Since the mammalian heart has negligible regenerative capacity, the death of large numbers of cardiomyocytes results in their replacement with a non-contractile, collagen-based scar that maintains the structural integrity of the ventricle and prevents catastrophic events, such as cardiac rupture (Frangogiannis, 2015).

Oxidative stress mediated by reactive oxygen species (ROS) plays a significant role in cardiomyocyte death and in the pathogenesis of HF, particularly after MI (Giordano, 2005; Hiroyuki et al., 2009). Several sources of increased ROS production have been identified in the overloaded heart, with the mitochondria being one of the major components (Laposky et al., 2006). Mammalian cells have developed multiple mechanisms to tightly regulate mitochondrial ROS levels. In addition to ROS scavengers as the first line of defense, inducible mitochondrial proton leak across the inner mitochondrial membrane, controlled by uncoupling proteins (Ucps), has emerged as an essential modulator of mitochondrial function (Akhmedov et al., 2015). Ucps can catalyze a regulated proton leak across the inner mitochondrial membrane, diverting free energy from the ATP synthesis chain to heat production, thus reducing ROS production (Brand et al., 2004; Casteilla et al., 2010). To date, five Ucp isoforms have been identified in mammals, of which Ucp3 is predominantly expressed in the heart (Bézaire et al., 2007).

Mice that lack Ucp3 have a larger infarct size after ischemia/reperfusion (I/R) than wild-type mice. Moreover, Ucp3 knockout hearts generate more ROS than wild-type hearts (Ozcan et al., 2013). Embryonic fibroblasts and adult cardiomyocytes from Ucp3 knockout mice show mitochondrial dysfunction, increased ROS production, and apoptotic cell death when compared with equivalent wild-type cells during *in vitro* hypoxia (Perrino et al., 2013). Moreover, pharmacologically upregulating Ucp3 can protect against I/R injury (Song et al., 2016). Because Ucp3 is a regulator of these processes in the HF setting, it could be used as a potential therapeutic target for the treatment of HF (Akhmedov et al., 2015). The methods currently found to increase the expression of Ucp3 include growth hormone stimulation, exposure to a cold environment, or fasting (Blaylock et al., 2004; Hayashi et al., 2018). In addition, HNE, WY-14643, and TZDs have also been found to upregulate Ucp3 (Matsuda et al., 1998; Lopez-Bernardo et al., 2015; Song et al., 2016). However, these drugs have obvious side effects, and their clinical applications are facing challenges (Tolman and Chandramouli, 2003; Cunningham, 2007; Grey, 2008; Lopez-Bernardo et al., 2015).

Honokiol (HK), a small-molecular-weight natural biphenolic compound extracted from the bark of magnolia trees, with the chemical structure 2-(4-hydroxy-3-prop-2-enyl-phenyl)-4-prop-2-enylphenol, has been reported to possess analgesic, anti-inflammatory, anti-oxidative, anti-tumor, and neuroprotective properties (Xianhe et al., 2003; Fried and Arbiser, 2009; Chen et al., 2011; Woodbury et al., 2013). It has previously been observed that HK reduces oxidative stress and reverses cardiac hypertrophy by activating Sirt1 and Sirt3, respectively (Pillai et al., 2015; Bin et al., 2018). In this study, we report that HK upregulates Ucp3 levels in both cells and animals. We also demonstrate that the HK-mediated upregulation of Ucp3 protects against myocardial oxidative damage *in vitro* and *in vivo*. To the best of our knowledge, this is the first report describing HK as an activator of Ucp3 and as a substance that ameliorates post-MI HF.

## Materials and Methods

### HK Emulsion Preparation

To prepare honokiol emulsion (HKE), 1 g of HK and 10 μl of vitamin E were dissolved in 30 ml soybean oil to form the oil phase. About 3 g of poloxamer 188 and 7 g of glycerol were dissolved in 30 ml of water to form the aqueous phase. The aqueous phase was slowly poured into the oil phase at 60°C, stirred at 1,400 rpm for 5 min, and adjusted to pH 8.0 with 0.1 M sodium hydroxide. The mixture was homogenized for 2 min at 200, 700, and 200 psi to obtain the final HKE.

### Animals

All animal studies were performed in accordance with the guidelines approved by the Institutional Animal Care and Use Committee of Sichuan University. Eight-week-old C57BL/6 mice were purchased from Beijing HFK Bioscience Co., Ltd. Ucp3 knockout C57BL/6 mice were purchased from Suzhou Cyagen Biosciences.

### Concentrations of HK in Plasma and Heart

Thirty-two mice were randomly divided into two groups and injected intraperitoneally (IP) with HK dissolved in soybean oil or HKE at a dose of 20 mg/kg. At 0.5, 1, 2, and 4 h after injection, the mice were sacrificed to collect plasma and hearts. Hearts were accurately weighed, homogenized in 0.5 ml deionized water with a tissue homogenizer, and extracted with methanol. Tissue homogenate extracts and plasma were analyzed using an ultra-fast liquid chromatography–triple quadrupole mass spectrometer system (LC/MS) (UFLC system, Shimadzu; AB5500 mass spectrometer, Applied Biosystems). The detailed method is provided in the Supplementary Data.

### Animal Model

After 1 week of acclimation, the mice were anesthetized with a mixture of ketamine and xylazine (100 and 2.5 mg/kg, respectively, IP injection). Under a dissecting microscope, animals were placed in the supine position on a heated operation board, and a midline cervical incision was made to expose the trachea. After successful endotracheal intubation, the cannula was connected to a volume-cycled rodent ventilator (Harvard) on room air with a stroke volume of 0.25 ml and respiratory rate of 130/min. After that, the mice were moved to the right lateral position, the chest cavity was accessed through the fourth intercostal space at the left sternal border through a small incision, and MI was produced by ligating the left anterior descending coronary artery with an 7-0 prolene suture at the site of the emergence of the vessels past the tip of the left atrium. Sham-operated animals underwent the same procedure at the same time without the ligation of the left coronary artery. After surgery, the mice were administered either HKE (20 mg/kg per day, qod) or blank emulsion intraperitoneally.

### Echocardiography

After 4 weeks of treatment, echocardiography was conducted using VIVIDi ultrasound (GE) and its corresponding probe (i12L-RS) with a center frequency of 13 MHz; animals were anesthetized with isoflurane. The left ventricular internal diameters during systole and diastole (LVIDs, LVIDd) were measured by M-mode echocardiography at the left parasternal short axis section, and then the EF and fractional shortening (FS) were calculated. Three cardiac cycles were recorded for each measurement.

### Masson’s Trichrome Staining

Myocardial fibrosis was assessed by Masson’s trichrome staining. Mouse hearts were excised and fixed in 4% paraformaldehyde, embedded in paraffin, and cut into 5 μm thick serial sections. Masson’s trichrome staining was performed on each section. Slice images were captured using Pannoramic 250/MIDI (3D HISTECH). The pathological sections were analyzed using Caseviewer (2.3 version).

### Reactive Oxygen Species Detection

The hearts were snap-frozen in Tissue-Tek OCT (Sakura) and stored at −80°C. Frozen tissue was cut by Cryotome E Frozen slicer (Thermo) into 8 μm thick sections and mounted on Superfrost/Plus slides. The unfixed tissue slides were incubated with dihydroethidium (DHE) (1:200, Servicebio) in PBS at room temperature for 30 min. The slides were then washed, fixed, mounted, and subjected to fluorescence microscopic analysis (Nikon Eclipse C1). The fluorescence intensity of the cardiac sections was measured using ImageJ software. The results were expressed as the mean fluorescence intensity.

### Primary Cultures of Cardiomyocytes

Primary cardiac myocyte cultures were prepared from neonatal mouse hearts. In brief, hearts were removed from 1–3-day-old pups and kept in cold PBS. The ventricles were cut into four-to-six evenly sized pieces using small scissors and digested using 0.25% trypsin at 4°C overnight. Digestion was continued five times with collagenase type II until no tissue chunks were visible. The tissue digest was spun, and the pellet was dissolved in DMEM/F12 (GIBCO) with 10% FBS (GIBCO) and 5% penicillin-streptomycin (GIBCO). Cells were pre-plated for 1 h to remove fibroblasts, and unattached cardiomyocytes in suspension were collected and plated in culture plates. Cardiomyocyte cultures were used after 24 h of plating.

### Mitochondrial Membrane Potential Measurement

The cells were cultured in a 6-well dish for 12 h in the presence or absence of 40 μM HK, and then, the medium was replaced with a normal medium containing 50 μM hydrogen peroxide for 12 h to induce oxidative damage. Mitochondrial membrane potential (MMP) was measured using a JC-1 MMP detection kit (Solarbio), as described in the instruction manual, and images were taken with a fluorescence microscope.

### RNA Sequencing and Transcriptomic Data Analysis

Total RNA was extracted from the hearts, and the mRNA was enriched with magnetic beads with Oligo (dT). mRNA was fragmented into short fragments to be used as templates to synthesize one-strand cDNA, and double-stranded cDNA was synthesized. A tail and sequencing linker was added to the purified double-stranded cDNA, and the fragment size was selected. The library was qualified using the Agilent 2100 Bioanalyzer and sequenced using an Illumina HiSeqTM 2500.

The expression of protein-coding genes was calculated using the FPKM method with StringTie2 software (version 1.3.3b). DESeq software (Version 1.18.0) was used to normalize the counts of each sample gene. The base mean value was used to estimate the expression and calculate multiple differences. The negative binomial distribution test was used to test the significance of differences in the reads. Multiples of difference and significance tests were used to screen for differentially expressed protein-coding genes. The default screening difference was *p* < 0.05, and the difference multiple was greater than two (https://dataview.ncbi.nlm.nih.gov/object/PRJNA737169?reviewer=9143j7dlacotrcni61d2vra5kg).

### Cell Culture

H9c2 cardiomyocytes and HEK293 cells were purchased from ATCC and cultured in high-glucose DMEM (GIBCO) supplemented with 10% fetal bovine serum (GIBCO) and 1% penicillin/streptomycin (GIBCO) at 37°C in a humidified atmosphere with 5% CO_2_.

### Quantitative Real-Time PCR

Total RNA was isolated from mouse hearts or cardiomyocytes using the Total RNA Isolation Kit (Foregene). The quality of the total RNA was determined by agarose gel electrophoresis. About 1μg of total RNA was reverse-transcribed using RT Easy^TM^ II (Foregene). The resultant cDNA was diluted 10-fold before PCR amplification. The reverse transcriptase minus reaction served as a negative control. mRNA levels were measured using SYBR green real-time PCR. The primer sequences of genes used for qRT-PCR analysis are listed in Supplementary Table S1.

### Western Blot

The heart tissue was homogenized in RIPA lysis buffer (Cwbiotech) and supplemented with 1 mM PMSF (Sigma) and 1× protease/phosphatase Inhibitor Cocktail (Cwbiotech). After quantification, each sample was denatured in SDS–polyacrylamide gel electrophoresis (PAGE) Loading Buffer (Cwbiotech) and boiled for 5 min. A total of 50 μg of protein was loaded in 10% SDS-PAGE and then transferred to a PVDF membrane (Millipore). After blocking in TBST solution with 5% non-fat milk for 2 h at room temperature, membranes were incubated overnight at 4°C with primary antibodies: anti-UCP3 (Zen-bio, 516996, 1:1,000) and anti-GAPDH (Abways, AB0036, 1:5,000). Membranes were then washed and incubated with species-specific HRP-conjugated secondary antibodies (Abways, AB0101, 1:5,000) at 1:5,000 in TBST with 5% non-fat milk for 1 h at room temperature. The blot was visualized by adding Superlumia ECL Plus HRP substrate kit (Abbkine) and then captured and analyzed by UVP Bio Imaging Systems (Sagecreation).

### Apoptosis Assay

Apoptosis assays were used to detect the protective effect of HK on hydrogen peroxide damage in cardiomyocytes. Cells were cultured in a 6-well dish for 12 h in the presence or absence of 40 μM HK, and the medium was then replaced with normal medium containing 100/400 μM hydrogen peroxide for 12 h to induce oxidative damage. The Annexin V-FITC/PI Apoptosis Detection Kit (Meilunbio, China) was used for the apoptosis assay, and a total of 8,000 cells were collected for each sample. All steps were performed in accordance with the manufacturer’s instructions.

### Statistical Analysis

Statistical analysis was performed using GraphPad Prism 6 software (version 6.01). All data were presented as the mean ± SD. The statistical significance of differences was determined by a Student t test between two groups or one-way ANOVA followed by Tukey’s multiple comparisons test for post hoc t test. Statistical significance was set at *p* < 0.05.

## Results

### HKE Improves Cardiac Function in Post-MI HF

**FIGURE 1:**
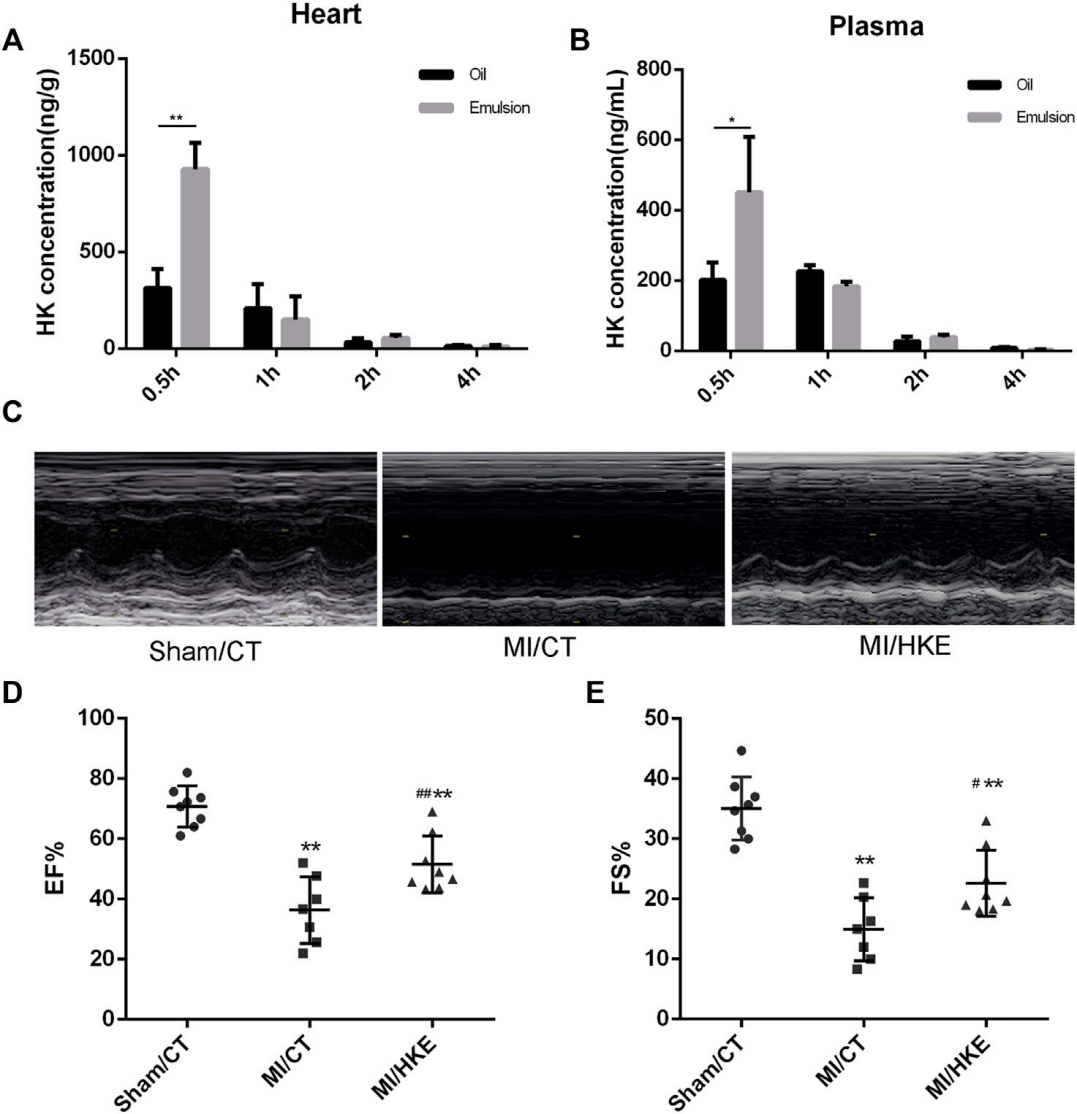
HKE improves cardiac function in post-MI HF. HKE or soybean oil dissolved HK were injected IP (20 mg/kg), and the concentration of HK in heart **(A)** and plasma **(B)** were monitored for 0.5, 1, 2, and 4 h after injection (*n* = 4 per group). Four weeks after MI, EF and FS were determined by echocardiography **(C–E)** (*n* = 7 or 8 per group). Values are shown as means ± SD, *p < 0.05, **p < 0.01 vs. Sham/CT group, #*p* < 0.05, ##*p* < 0.01 vs. MI/CT group.

To obtain better intake, HK was prepared as an emulsion (HKE). We compared the tissue distributions of oil-soluble HK and HKE. Half an hour after administration, the average cardiac concentration of oil-soluble HK and HKE groups was 313.4 and 928.4 ng/g, respectively (Figure 1A). Similar results were observed in plasma; the average plasma concentration of HK in the HKE group was 451.1 ng/ml, which was significantly higher than that in the oil-soluble HK group (201.6 ng/ml) (Figure 1B). Moreover, the HK concentration in the HKE group did not decrease faster than that of the oil-dissolved group over time, demonstrating that HKE improved its absorption and distribution in the heart.

To evaluate the therapeutic effect of HKE in post-MI HF, mice were IP injected with HKE (20 mg/kg per day, qod), according to our previous dose investigation (Yang et al., 2017), or the same volume of blank emulsion immediately after MI. After 4 weeks of treatment, cardiac function was examined using echocardiography. Compared to the sham group, EF and FS in the MI/CT group were markedly reduced. Strikingly, in comparison with the MI/CT group, the MI/HKE group showed a significant increase in EF and FS (Figures 1C–E). Moreover, the prolonged survival rate of the MI/HKE group was also observed in comparison with that of the MI/CT group (Supplementary Figure S1).

### HKE Inhibits Myocardial Remodeling, Reduces the ROS, and Alleviates Myocardial Fibrosis Caused by MI

**FIGURE 2:**
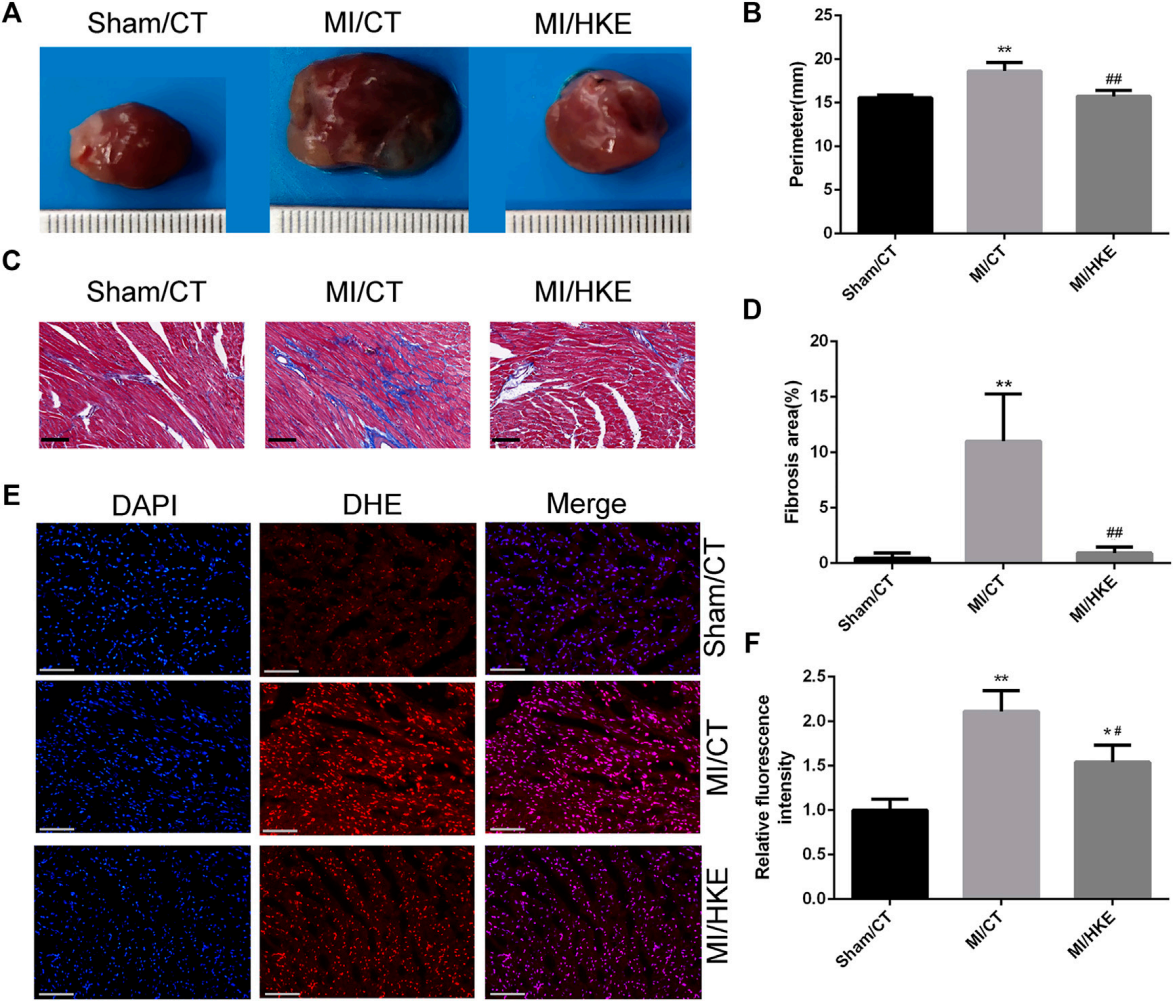
HKE inhibits myocardial remodeling, reduces the ROS, and alleviates myocardial fibrosis *in vitro*. Typical heart appearance in groups Sham/CT, MI/CT, and MI/HKE **(A)**. The perimeter of the largest part cross section of the heart was measured to evaluate myocardial remodeling **(B)**. Masson’s Trichrome staining was conducted to evaluate myocardial fibrosis, the fibrotic area was indicated as a blue region, the red frame encircles the fibrosis away from the infarct area in the MI group and similar locations in other groups, scale bar: 50 μm **(C)**. The fibrosis of red frame area was measured using ImageJ software **(D)** (n = 3 per group). Heart slides were stained with DHE to analyze the production of ROS *in situ*, scale bar: 100 μm **(E)**. The fluorescence intensity was measured using ImageJ software **(F)** (*n* = 3 per group). Values are shown as means ± SD, *p < 0.05, **p < 0.01 vs. Sham/CT group or control, ^*#*^ *p* < 0.05, ^*##*^ *p* < 0.01 vs. MI/CT.

To further investigate the cardioprotective role of HKE, cardiac remodeling, myocardial fibrosis, and cardiac ROS were examined. The hearts of the MI groups were significantly larger than the sham group, and HKE effectively reduced the heart size caused by MI (Figures 2A,B). Myocardial fibrosis causes left ventricular dysfunction and promotes HF (Gonzalez et al., 2018). Masson staining showed that HKE relieved myocardial fibrosis near the infarcted area (Figures 2C,D, Supplementary Figure S2). Cardiac ROS was examined *in vivo* by using DHE fluorescence as a marker. Compared with the sham/CT group, greatly increased DHE fluorescence was observed in the MI/CT group. The HKE treatment significantly reduced the DHE fluorescence in the MI animals (Figures 2E,F).

### HK Relieves the Abnormality of MMP and Protects Against the Apoptosis Caused by Peroxide *in vitro*

**FIGURE 3:**
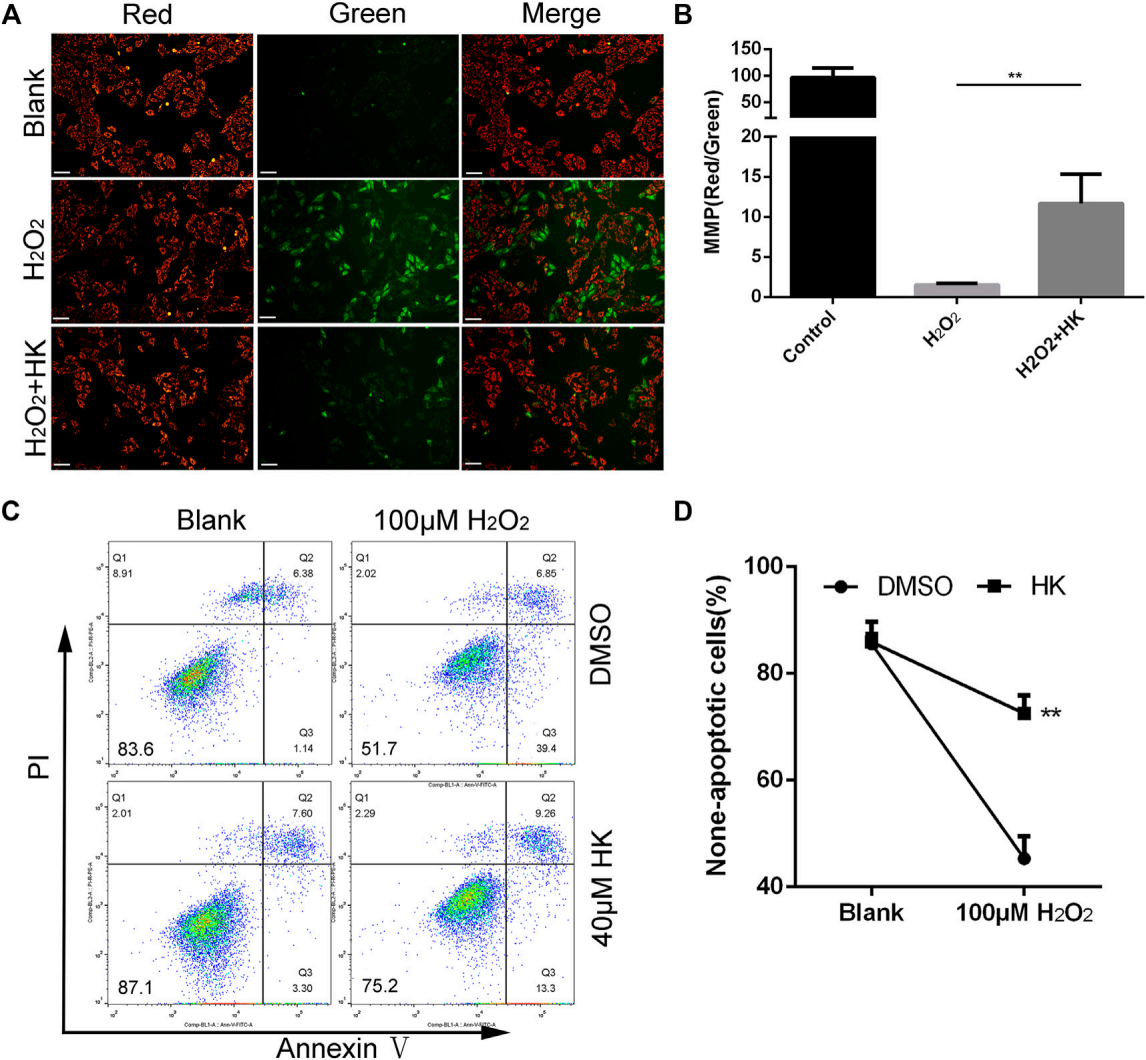
HK normalizes MMP and protects against the apoptosis caused by peroxide *in vitro.* For MMP detection, primary cardiomyocytes from neonatal mice hearts were cultured in a six-well dish for 12 h in the presence or absence of 40 μM HK, the medium was then replaced with normal medium containing 50 μM hydrogen peroxide for 12 h to induce oxidative damage. Cardiomyocytes were stained with JC-1 to monitoring the MMP *in vitro*, scale bar: 100 μm **(A)**. The fluorescence intensity was measured using ImageJ software **(B)** (*n* = 3 per group). For apoptosis detection, cardiomyocytes were cultured in six well dish for 12 h in the presence or absence of 40 μM HK, the medium was then replaced with normal medium containing 100 μM hydrogen peroxide for 12 h to induce oxidative damage. Annexin V-FITC/PI Apoptosis Detection Kit were used for apoptosis assay by FACS analysis, data show quantification of non-apoptotic cells **(C,D)** (*n* = 3 per group). A total of 8,000 cells were collected for each sample. Values are shown as means ± SD. *p < 0.05, **p < 0.01 vs. H_2_O_2_ treatment group.

HK with a minimum of 40 μM could significantly relieve the oxidative damage in H9c2 cells (Supplementary Figure S3). Therefore, this concentration was used in the following *in vitro*. Abnormal MMP is directly related to an increase in ROS levels (Korshunov et al., 1997). HK maintains MMP in different cells (Wang et al., 2017; Zheng et al., 2018; Ye et al., 2019). JC-1 staining was used to monitor MMP in neonatal mouse cardiomyocytes. HK can significantly relieve the abnormality of cardiomyocyte MMP caused by 50 μM hydrogen peroxide (Figures 3A,B).

In addition, 100 μM hydrogen peroxide was used to induce apoptosis in neonatal mouse cardiomyocytes. The proportion of non-apoptotic cells in cardiomyocytes treated with HK was significantly higher than that in cardiomyocytes without HK. (Figures 3C,D).

Collectively, these results show that HK can relieve the abnormality of MMP and protect against apoptosis caused by peroxide in neonatal mouse cardiomyocytes.

### RNAseq and Differential Gene Expression Analysis

It has previously been observed that HK reduces oxidative stress and reverses cardiac hypertrophy by activating Sirt1, Sirt3, and Nrf2, respectively (Pillai et al., 2015; Bin et al., 2018). However, in the MI model, no transcriptional level change was observed in *Sirt1*, *Sirt3,* and *Nrf2* after HKE administration (Supplementary Figure S4).

**FIGURE 4:**
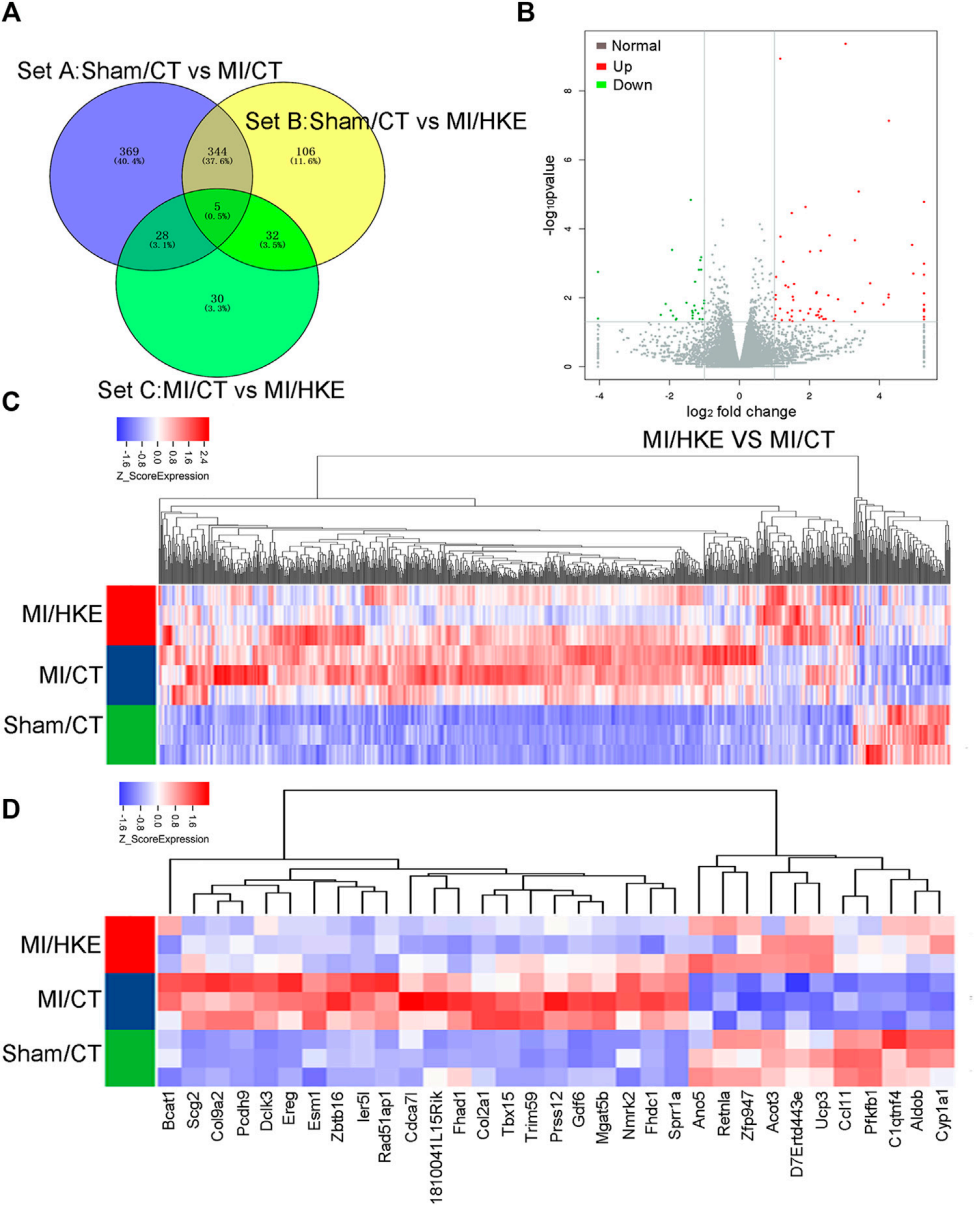
RNAseq and differential gene expression analysis. Total RNA was isolated from heart tissue for RNAseq (*n* = 3 per group). The different expressed gene numbers between each group were shown by Venn diagram **(A)**. The volcano plot shows the different expressed genes between MI/CT and MI/HKE **(B)**. Cluster analysis of all differential genes **(C)**. Cluster analysis of 33 differential genes in the intersection of Set A and Set C **(D)**.

To reveal the molecular mechanism of the protective effect of HK on post-MI HF, RNAseq was performed (Figure 4, Supplementary Figure S5). The screening criteria were a fold-change >2 and *p* < 0.05. There were 746 differentially expressed genes between the Sham/CT and MI/CT groups (Set A), 487 between the Sham/Blank and MI/HKE groups (Set B), and 95 between MI/CT and MI/HKE groups (Set C). Among the 95 genes in Set C, 67 genes were upregulated and 28 genes were downregulated after HKE treatment (Figures 4A–C). GO annotation showed that these genes were related to the ion channels and membrane potentials (Supplementary Table S2). Genes at the intersection of Set A and Set C, and differences between MI/Blank and the other two groups, had similar expression levels between Sham/CT and MI/HKE groups (Figure 4D). These genes have been shown to be restored after HKE administration and may be key factors for post-MI HF treatment.

### HKE Restores Fibrosis-Related Genes and Ucp3

**FIGURE 5:**
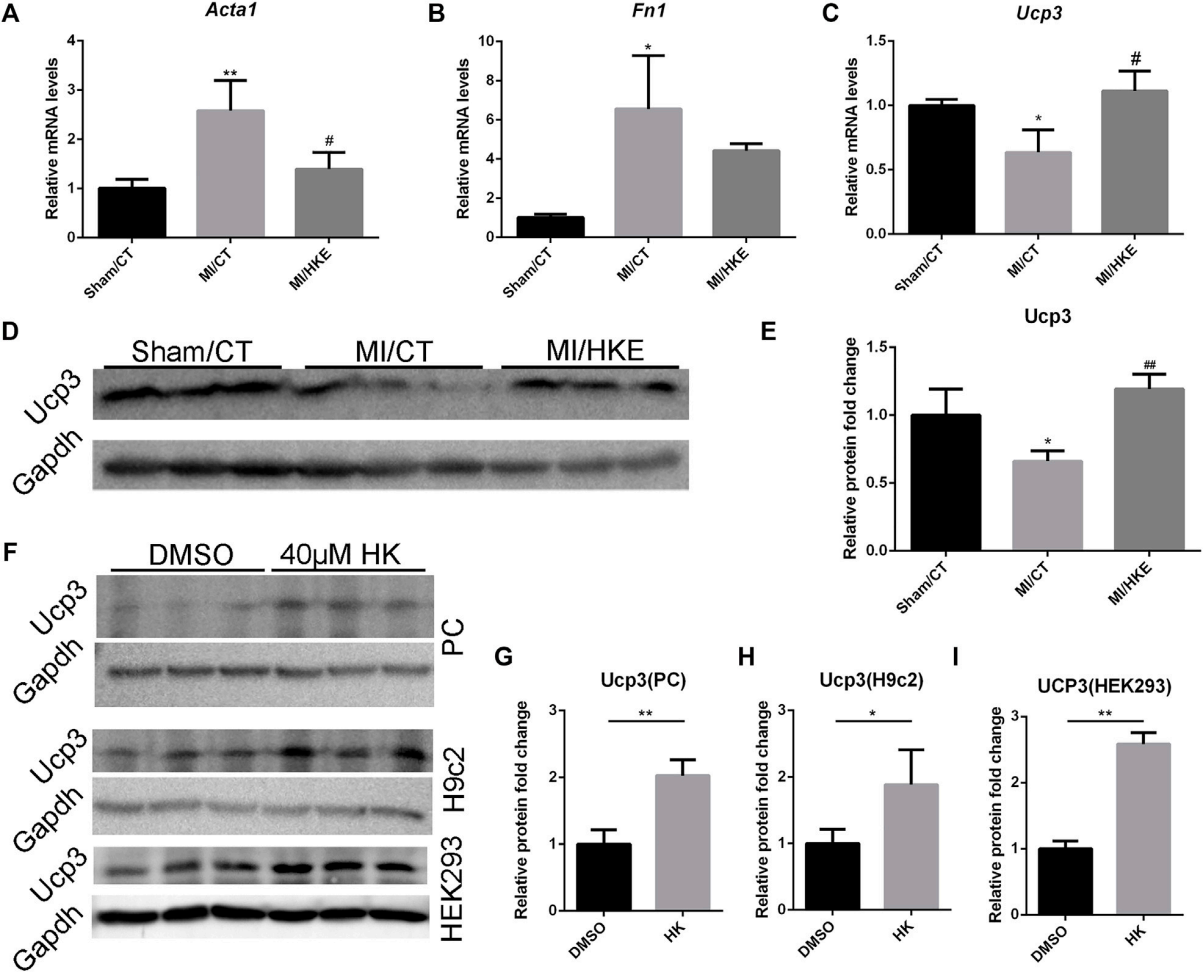
HKE restores fibrosis-related genes and Ucp3. The mRNA expression of *Acta1, Fn1*, and *Ucp3* in myocardial tissue was detected using real-time PCR (*n* = 3 per group). 2^-△△Ct^ method was used to analyze relative gene expression levels **(A–C)**. The Ucp3 abundance in myocardial tissue were detected by Western blot. Gapdh was included as a loading control (*n* = 3 per group) **(D,E)**. After treatment with 40 μM HK or equal volume of DMSO for 24 h, The Ucp3 abundance in primary cardiomyocytes (PC), H9c2 and HEK293 were detected by Western blot. Gapdh was included as a loading control (*n* = 3 per group) **(F–I)**. Values are shown as means ± SD, *p < 0.05, **p < 0.01 vs. Sham/CT or DMSO, ^*#*^ *p* < 0.05, ^*##*^ *p* < 0.01 vs. MI/CT group.

Through the screening of sequencing results, we found that HKE restored the expression of fibrosis markers Acta1 (Schwartz et al., 1986) and Fn1 (Knowlton et al., 1992), which were verified by qRT-PCR (Figures 5A,B). In addition, 12 collagen-coding genes, which are also fibrosis markers (Ding et al., 2020), underwent significant changes after HKE intervention (Supplementary Figure S6). Together, these results provide important insights into the molecular mechanism by which HK alleviates myocardial fibrosis.

We also found some genes related to heart function in the differentially expressed genes after HK intervention (Supplementary Table S3). Among them, Ucp3, a proton-transporter protein, is directly related to MMP, ROS, and cardioprotection (Ozcan et al., 2013; Perrino et al., 2013). Interestingly, these characteristics are consistent with the above-mentioned effects of HK on MMP maintenance, oxidative damage resistance, and cardioprotection. In addition, at least one group had an average FPKM >3 (Supplementary Table S4). Therefore, we hypothesized that Ucp3 may play a key role in the protection of HK against post-MI HF. qRT-PCR and Western blotting verified that HKE significantly restored the decrease in Ucp3 expression caused by MI (Figures 5C–E). In addition, we also observed the upregulation of Ucp3 by HK in neonatal mouse cardiomyocytes, rat H9c2 cardiomyocyte cell line, and human HEK293 cells *in vitro* (Figures 5F–I, Supplementary Figure S7).

### Ucp3 Mediates the Protection of HK Against Oxidative Damage in H9c2 Cardiomyocyte and Neonatal Mice Cardiomyocyte

**FIGURE 6:**
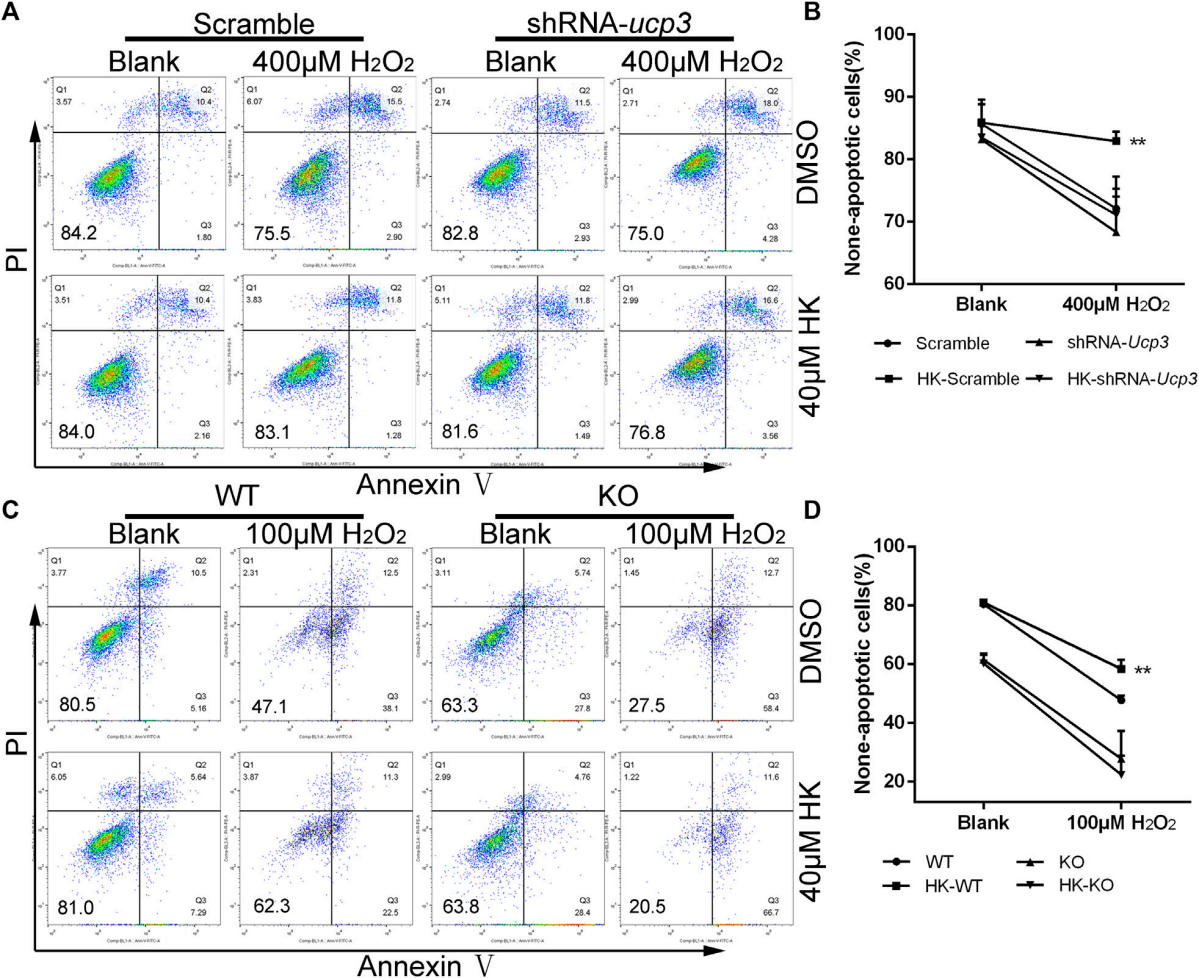
Ucp3 mediates the protection of HK against oxidative damage in cardiomyocyte H9c2 and neonatal mice cardiomyocyte. For apoptosis detection, Wild-type H9c2 and H9c2 cells with disturbed Ucp3 expression or primary cardiomyocytes from WT and Ucp3-KO mice were cultured in six well dish for 12 h in the presence or absence of 40μM HK, respectively. The H9c2 medium was then replaced with normal medium containing 400 mM hydrogen peroxide **(A,B)** and primary cardiomyocytes replaced with normal medium containing 100 mM hydrogen peroxide **(C,D)** for 12 h to induce oxidative damage. Annexin V-FITC/PI Apoptosis Detection Kit was used for apoptosis assay by FACS analysis, data show quantification of none-apoptotic cells (*n* = 3 per group). Values are shown as means ± SD. *p < 0.05, **p < 0.01 vs. 100/400 μM H_2_O_2_ treatment WT/Scramble group.

To confirm the hypothesis that HK can achieve cardioprotection by stimulating the expression of Ucp3, we first investigated whether the protective effect of HK against cellular oxidative damage depended on Ucp3 *in vitro*. Ucp3 knockdown with short hairpin RNA was created in the rat H9c2 cardiomyocyte cell line (Supplementary Figure S8). Wild-type and shRNA-Ucp3 H9c2 cells were treated with H_2_O_2_ in the presence or absence of HK. HK treatment caused H_2_O_2_-induced apoptosis in wild-type but not in Ucp3 knockdown cells, suggesting that the protective effect of HK is dependent on the upregulation of Ucp3 (Figures 6A,B).

To verify that the therapeutic effect of HK on post-MI HF is achieved by upregulating Ucp3, Ucp3 knockout mice were constructed. Using Ucp3 knockout and wild-type neonatal mouse cardiomyocytes, we confirmed that HK’s protection against cardiomyocyte damage caused by peroxide depends on Ucp3 *in vitro*. The results of FACS analysis also showed that HK only has a protective effect against oxidative damage in wild-type mouse cardiomyocytes (Figures 6C,D).

### The Protection of HKE Against Post-MI HF Depends on Ucp3

**FIGURE 7:**
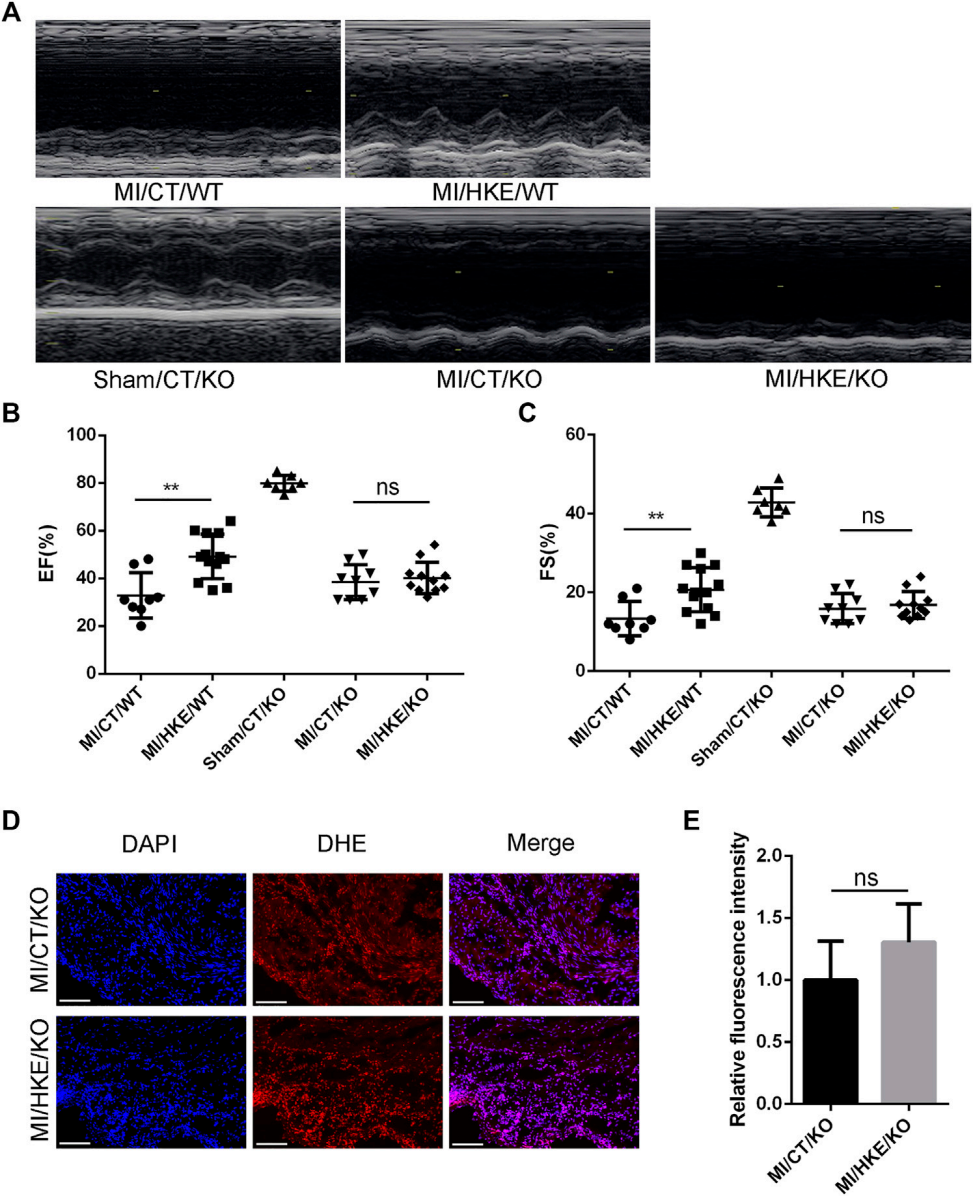
Ucp3 mediates the protection of HKE against post-MI HF. Construction of MI model with Ucp3 knockout and WT mice,4 weeks after MI, EF and FS were determined by echocardiography **(A–C)**. Heart slides were stained with DHE to analyze the production of ROS *in situ,* scale bar: 100 μm **(D)**. The fluorescence intensity was measured using ImageJ software **(E)** (*n* = 3 per group). Values are shown as means ± SD, *p < 0.05, **p < 0.01, ns: no significance.

Consistent with the *in vitro* results, HKE did not restore the damage to mouse heart function caused by MI in Ucp3 knockout mice (Figures 7A–C). In addition, HKE treatment did not reduce the level of ROS in the myocardium (Figures 7D,E).

## Discussion

In this study, we report that HK can increase Ucp3 levels. HK treatment attenuated H_2_O_2_-induced injury to cardiomyocytes *in vitro*, as well as ROS and post-MI HF *in vivo*. In addition, we showed that HKE treatment prevented the induction of cardiac fibrosis and reduced cardiac hypertrophy. To the best of our knowledge, this is the first report describing that HK can increase Ucp3 abundance, which is capable of inhibiting ROS, thus ameliorating post-MI HF.

HF is defined as the heart failing to pump enough blood to meet the body’s needs. HF mortality rates are high, with a 5-year survival rate of less than 50% once diagnosed. Due to modern medical advancements, the survival rates and survival times of patients with acute heart disease or congenital heart disease have greatly improved. Unfortunately, these factors directly lead to a dramatic increase in HF risk (Gurvitz et al., 2016; Ntiloudi et al., 2016). HF can be divided into HF with reduced ejection fraction (HFrEF) and HF with preserved ejection fraction (HFpEF), based on whether the EF value is affected (Tsao et al., 2018). As the underlying mechanism of HFpEF is currently unclear; no therapy has been proven to improve adverse outcomes in patients with HFpEF (Elgendy et al., 2019). Current medications for HFrEF include drugs that reduce afterload, such as β-adrenergic blockers, angiotensin-converting enzyme inhibitors, angiotensin-receptor blockers, isosorbide dinitrate, and hydralazine hydrochloride; drugs that reduce blood volume, such as aldosterone antagonists and diuretics; and drugs that increase cardiac contractility, improve cardiac pumping function, and slow heart rate, such as digoxin. However, none of these drugs can efficiently reverse HF (Leach and Martin, 2018). An improvement in the pathogenesis and development of new drugs is imperative.

HK is a small allyl-containing biphenol compound with the molecular formula C_18_H_18_O_2_, which has been extensively researched and developed because of its numerous applications and good safety. Due to poor water solubility, the oral absorption of HK is limited, and most studies use IP administration (Godugu et al., 2017). In this study, a lipid emulsion was used, which is a nanodrug carrier with good biocompatibility and the ability to incorporate poorly water-soluble drugs (Tamilvanan, 2004). The results show that HKE improves the absorption of HK and the content of HK in the heart, which laid a foundation for the therapeutic effect of HK on post-MI HF.

HK has a simple structure and exerts its effects on multiple molecular targets. It has been reported that HK is involved in numerous biological processes, such as apoptosis, EGFR signal transduction, STAT3 activation cascade, mTOR pathway, NF-κB signaling, autophagy, and cell cycle (Banik et al., 2019). However, there are few reports on the cardioprotective effects of HK. Lo YC and others showed that HK can protect rat heart mitochondria against lipid peroxidation in 1994 (Lo et al., 1994). The same team found that HK may protect the myocardium against ischemic intervention and suppress vascular arrhythmia during ischemia and reconstruction (Tsai et al., 1996). Pillai et al. reported that HK is a pharmacological activator of Sirt3, capable of blocking and reversing the cardiac hypertrophic response and protecting the heart from doxorubicin-induced cardiotoxicity (Pillai et al., 2015; Pillai et al., 2017). Recently, studies have shown that HK can protect the heart by protecting the mitochondria and inhibiting ROS (Huang et al., 2017; Zhang et al., 2018; Tan et al., 2019). In this study, we show that HK ameliorates post-MI HF through Ucp3, a mitochondrial inner membrane proton transporter, mediated by ROS inhibition. The inhibition of ROS and cardioprotection by HK were further confirmed both *in vitro* and *in vivo*. However, it is not clear how HK can increase the abundance of Ucp3. Bugge et al. suggested that PPARγ can directly bind to the enhancer of the first intron of Ucp3 to increase protein expression (Bugge et al., 2010). HK happens to be an agonist of PPARγ (Atanasov et al., 2013). Huang and others observed that HK protects the heart from Dox-cardiotoxicity *via* improving mitochondrial function, by not only repressing mitochondrial protein acetylation but also enhancing PPARγ activity in the heart (Huang et al., 2017). In view of all that has been mentioned so far, one may suppose that HK upregulates Ucp3 by activating PPARγ.

In response to other studies, we also found that HK can inhibit myocardial remodeling caused by MI, but the reason for this needs further research. Fortunately, several studies have provided some inspiration. Pillai et al. showed that HK enhances the expression of Sirt3 nearly twice in the TAC model, thereby inhibiting or even reversing myocardial hypertrophy (Pillai et al., 2015). However, we did not observe any changes in Sirt3 expression in the MI model. Singh et al. described that HK can inhibit class I histone deacetylases (HDACs) in non-small cell lung cancer cells (Singh et al., 2013). We also found that HK increased the expression of acetylated H3 and H4 in cardiomyocytes (data not shown). Interestingly, class I HDACs can promote pathological cardiac growth and impair cardiac function (Xie and Hill, 2013). Kee et al. show that HDAC2, a class I HDAC, is activated during cardiac hypertrophy (Kee et al., 2008). HDAC2 deficiency attenuates cardiac hypertrophy in mice by increasing the transcription of the gene encoding phosphatidylinositide phosphatase SAC2 (Trivedi et al., 2007). HDAC8 activity was elevated in the hypertrophied hearts of DOCA–salt hypertensive rats and reduced by treatment with valproic acid (Kee et al., 2013). The use of class I HDAC inhibitors has been shown to effectively alleviate myocardial remodeling (Trivedi et al., 2007; Gallo et al., 2008; Chen et al., 2015; Morales et al., 2016). Whether HK can reduce myocardial remodeling through class I HDAC inhibitor activity or not requires further research.

## Data Availability

The datasets presented in this study can be found in online repositories. The names of the repository/repositories and accession number(s) can be found below: BioProject, PRJNA737169.
