# Corrigendum: Honokiol ameliorates post-myocardial infarction heart failure through Ucp3-mediated reactive oxygen species inhibition

**DOI:** 10.3389/fphar.2022.1000887

**Published:** 2022-09-27

**Authors:** Jianyu Liu, Minghai Tang, Tao Li, Zhengying Su, Zejiang Zhu, Caixia Dou, Yan Liu, Heying Pei, Jianhong Yang, Haoyu Ye, Lijuan Chen

**Affiliations:** ^1^ State Key Laboratory of Biotherapy and Cancer Center, National Clinical Research Center for Geriatrics, West China Hospital of Sichuan University, Chengdu, China; ^2^ West China-Washington Mitochondria and Metabolism Center, Department of Anesthesiology, Laboratory of Anesthesiology and Translational Neuroscience Center, West China Hospital of Sichuan University, Chengdu, China

**Keywords:** heart failure, myocardial infarction, honokiol, reactive oxygen species, UCP3

In the published article, there was an error in the **Funding** statement. “This work was supported by National Key Programs of China during the 13th Five-Year Plan Period (2018ZX09711001-002-012) and 1.3.5 project for disciplines of excellence, West China Hospital, Sichuan University (ZY2017202).” The correct **Funding** statement appears below.

Funding

“This work was supported by National Key Programs of China during the 13th Five-Year Plan Period (2018ZX09711001-002-012) and 1.3.5 project for disciplines of excellence, West China Hospital, Sichuan University (ZYGD20001).”

The authors apologize for this error and state that this does not change the scientific conclusions of the article in any way. The original article has been updated.

