# Supplementary figures and images for: Honokiol Ameliorates Post-Myocardial Infarction Heart Failure Through Ucp3-Mediated Reactive Oxygen Species Inhibition
Source: Front Pharmacol. 2022 Feb 21;13:811682. doi: 10.3389/fphar.2022.811682 (PMC8899544; doi:10.3389/fphar.2022.811682)

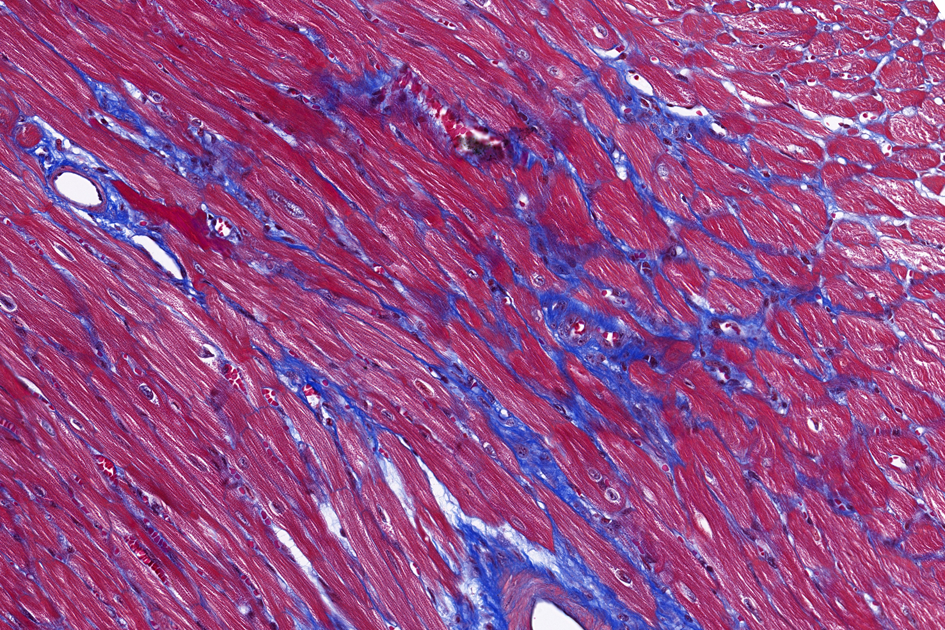

Supplement: Supplementary file 1 [file DataSheet1.ZIP › original source data1/fig2/C/MI-CT_20.0x.tif]

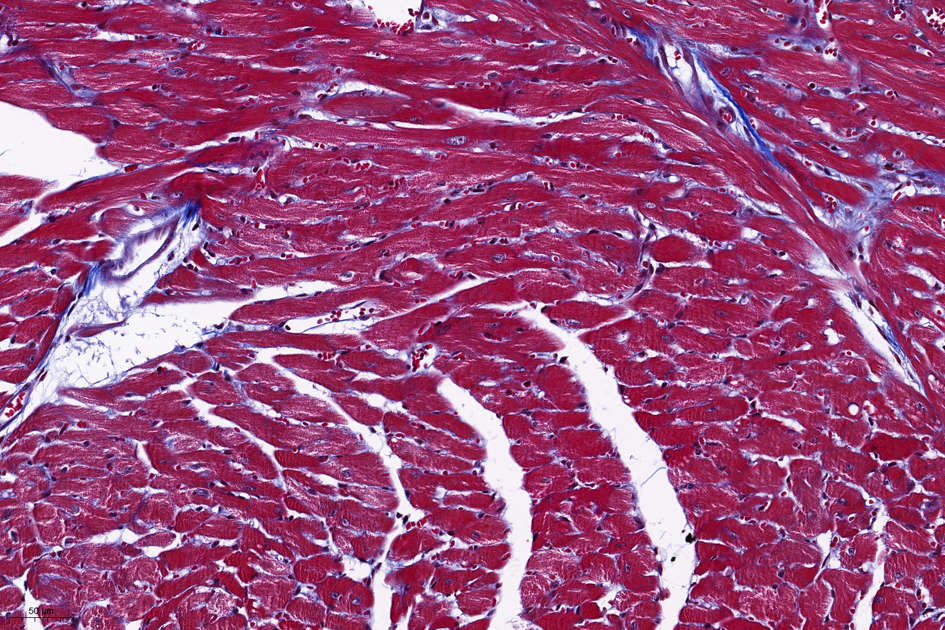

Supplement: Supplementary file 1 [file DataSheet1.ZIP › original source data1/fig2/C/MI-HKE_20.0x.tif]

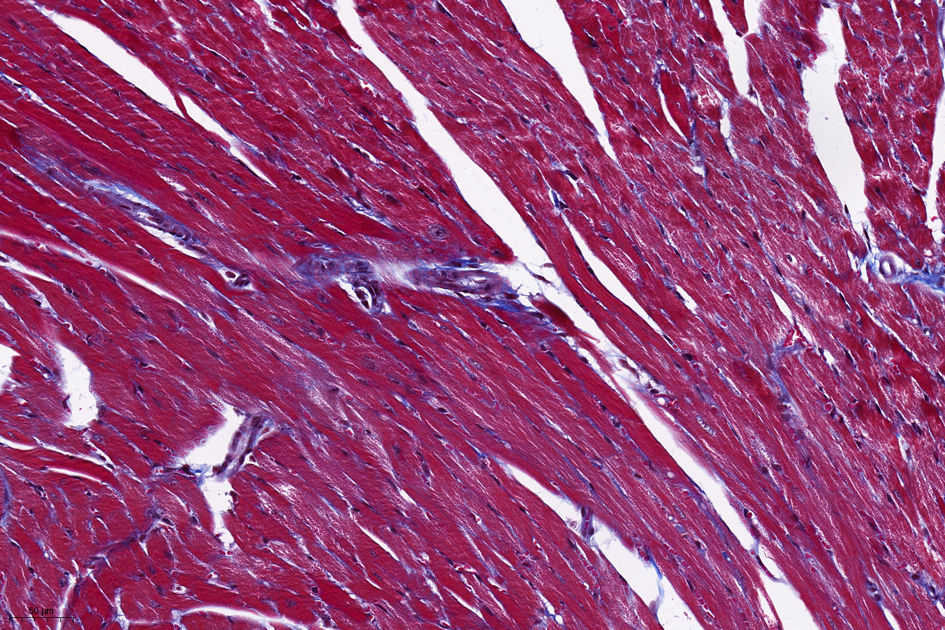

Supplement: Supplementary file 1 [file DataSheet1.ZIP › original source data1/fig2/C/Sham-CT_20.0x.tif]

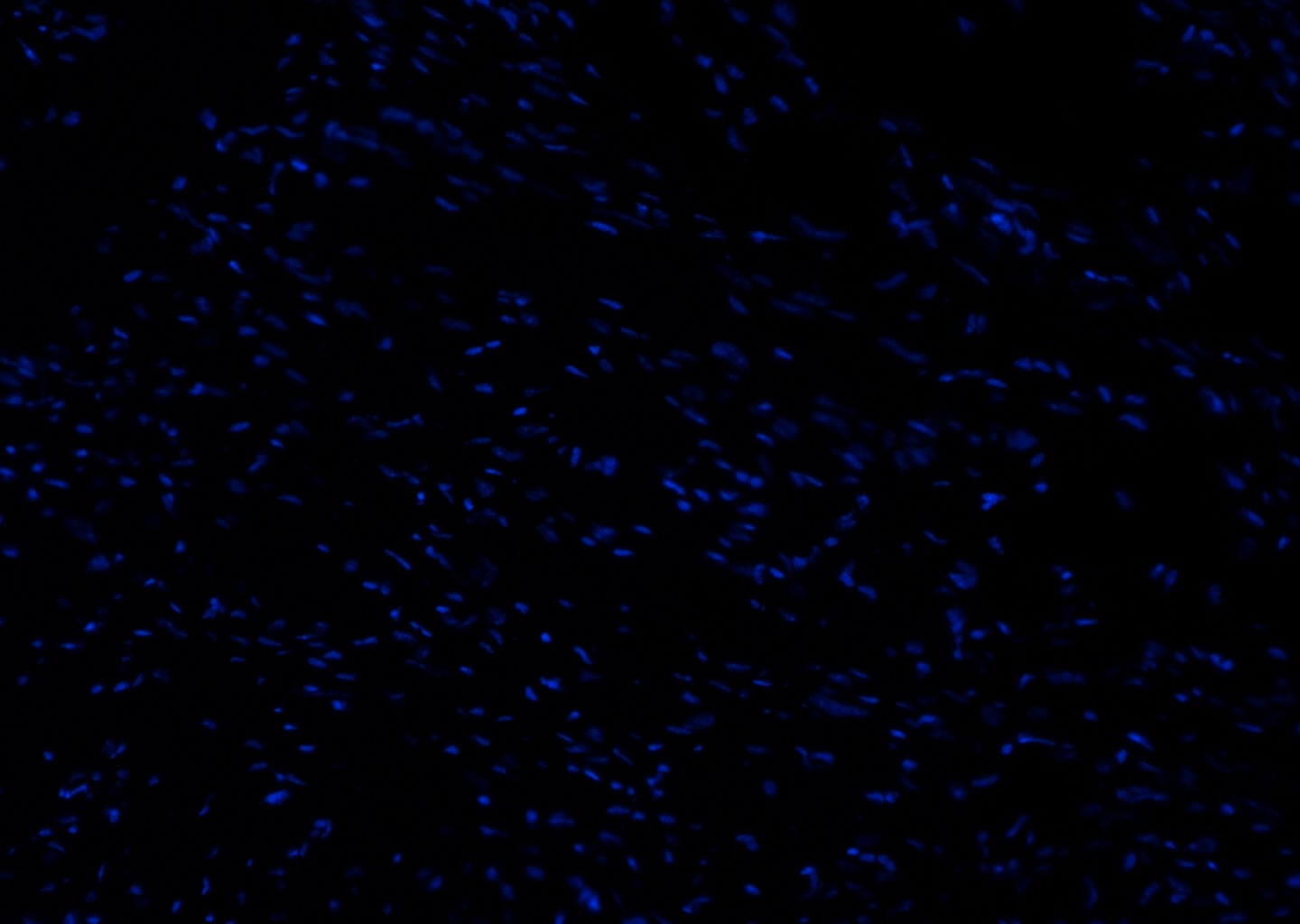

Supplement: Supplementary file 1 [file DataSheet1.ZIP › original source data1/fig2/E/MI-CT-DAPI.jpg]

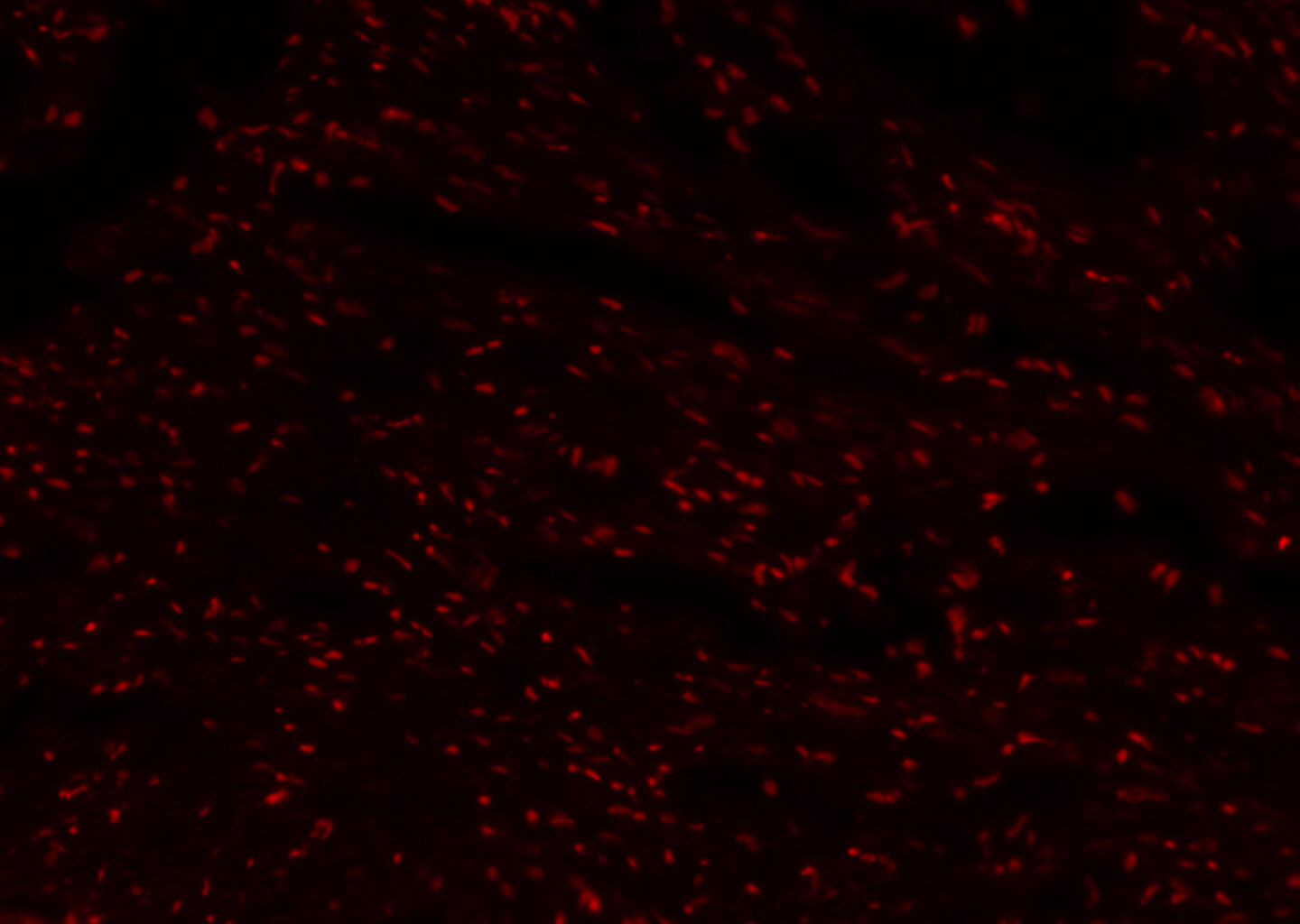

Supplement: Supplementary file 1 [file DataSheet1.ZIP › original source data1/fig2/E/MI-CT-DHE.jpg]

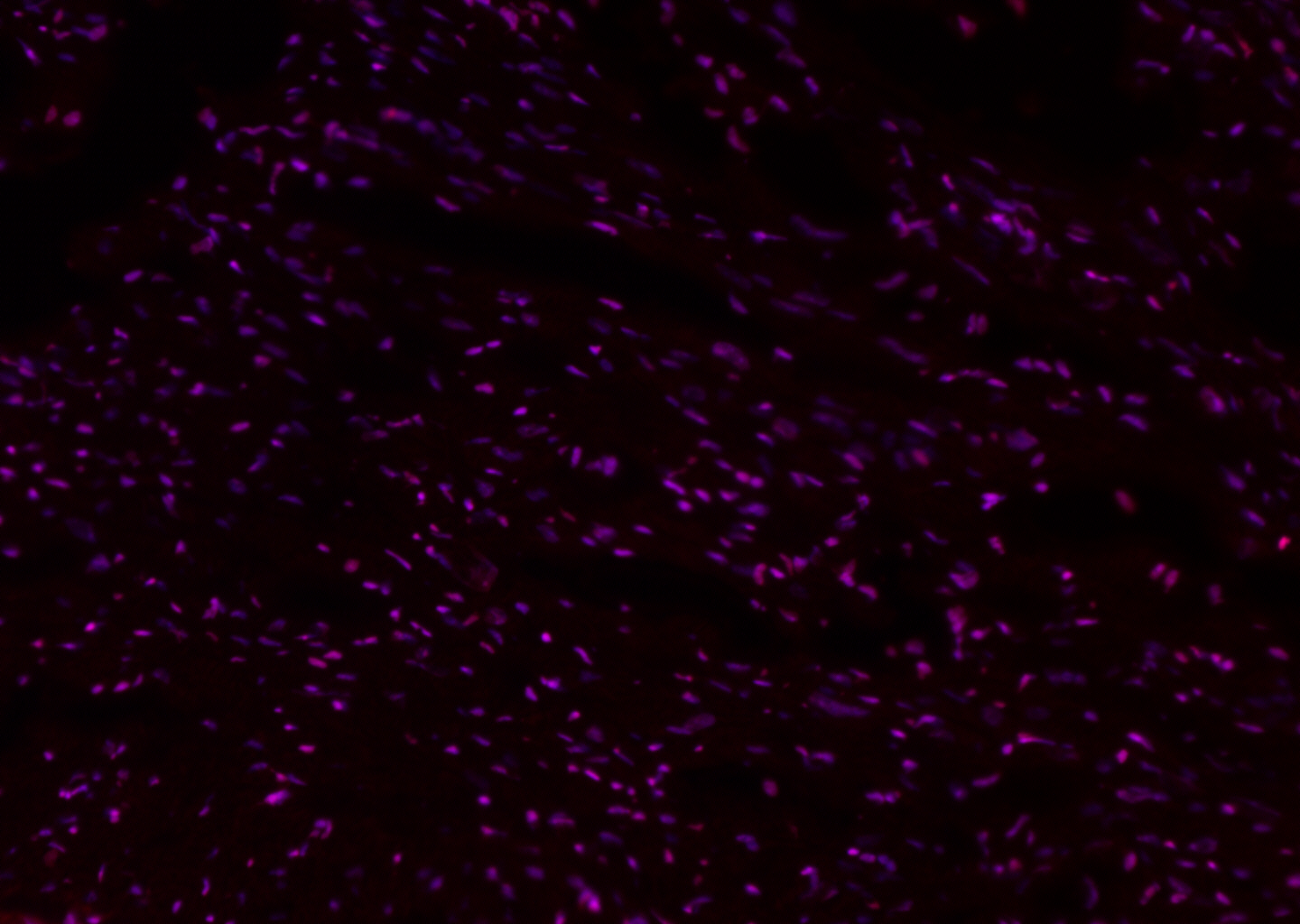

Supplement: Supplementary file 1 [file DataSheet1.ZIP › original source data1/fig2/E/MI-CT-merge.jpg]

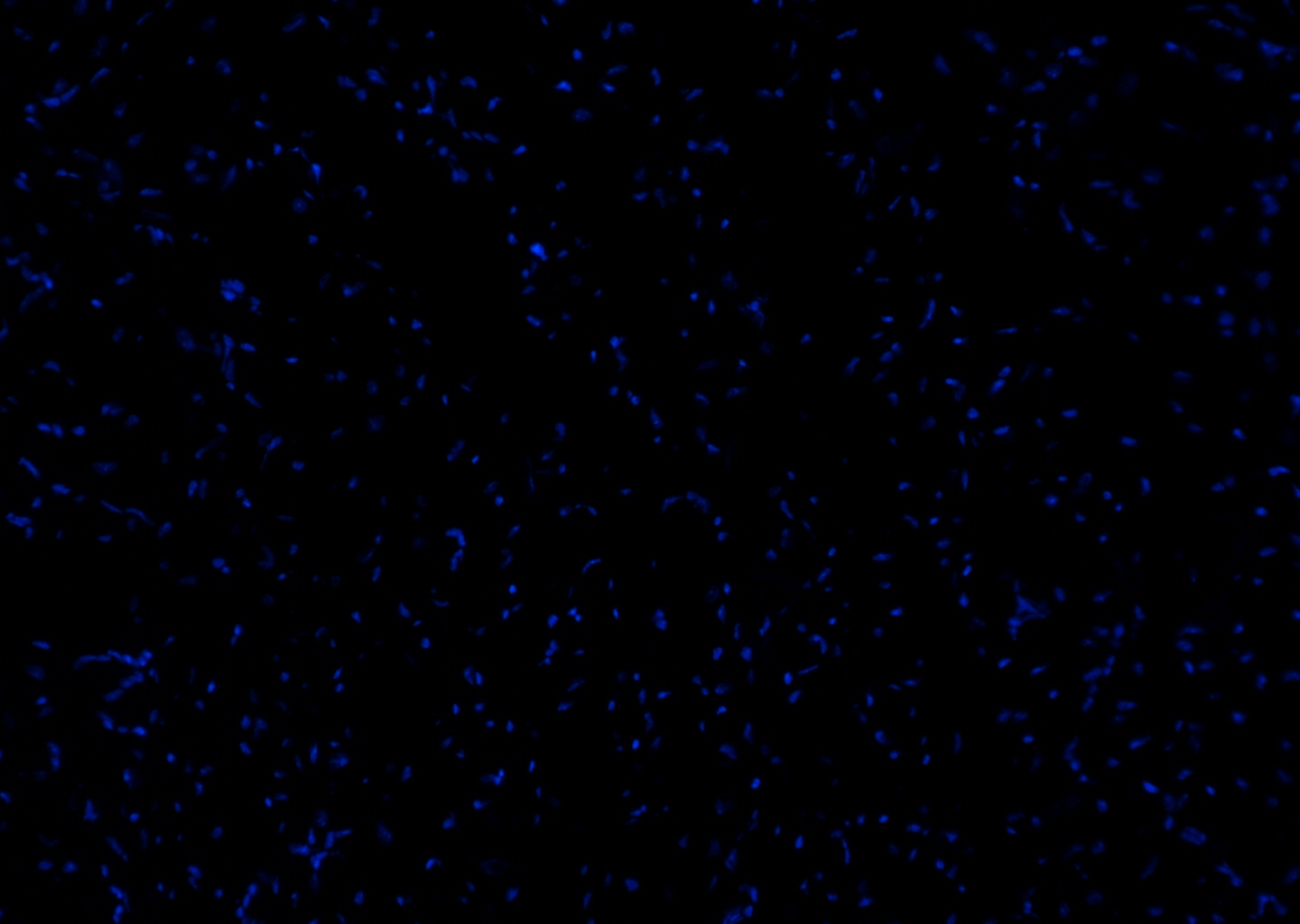

Supplement: Supplementary file 1 [file DataSheet1.ZIP › original source data1/fig2/E/MI-HKE-DAPI.jpg]

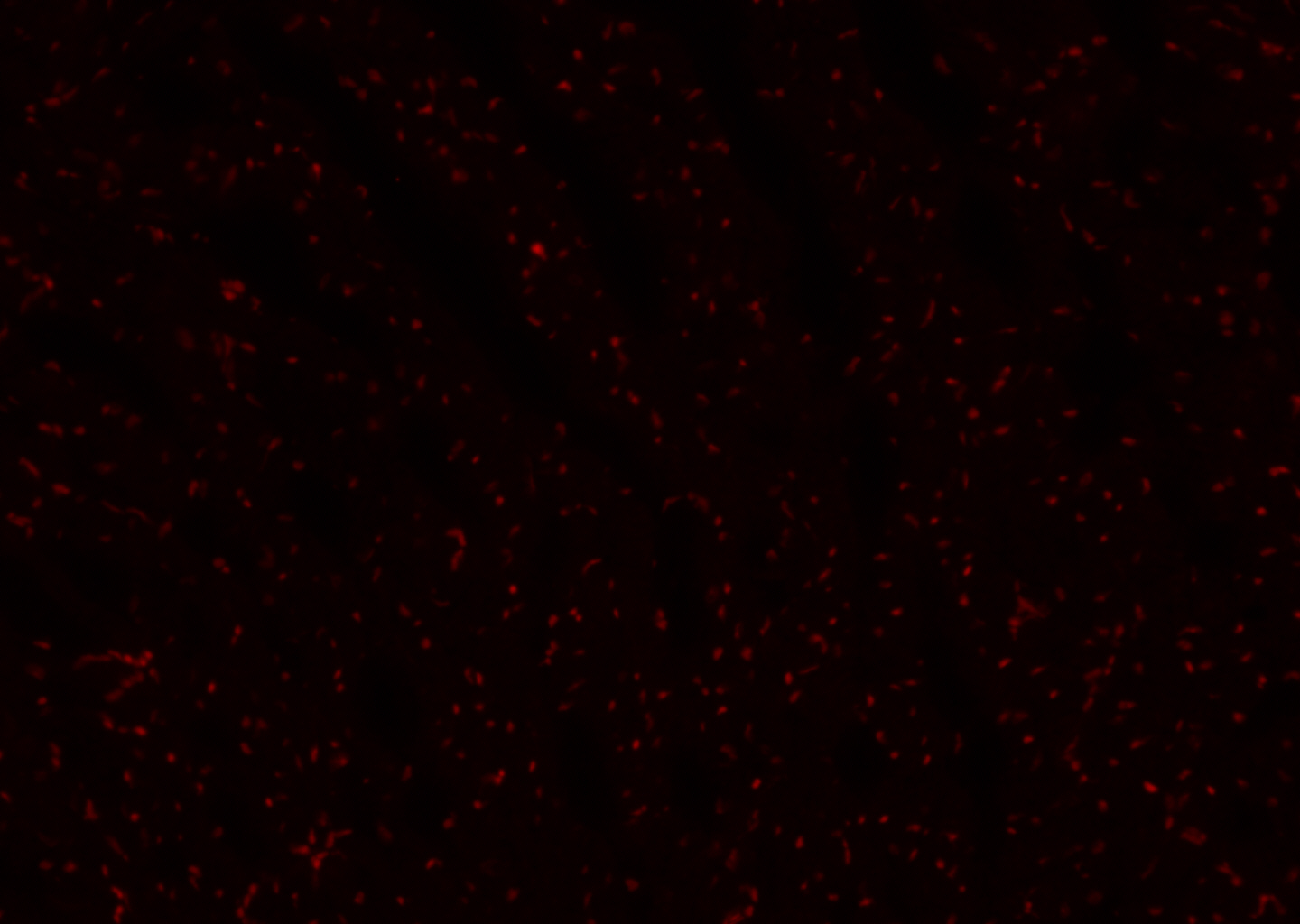

Supplement: Supplementary file 1 [file DataSheet1.ZIP › original source data1/fig2/E/MI-HKE-DHE.jpg]

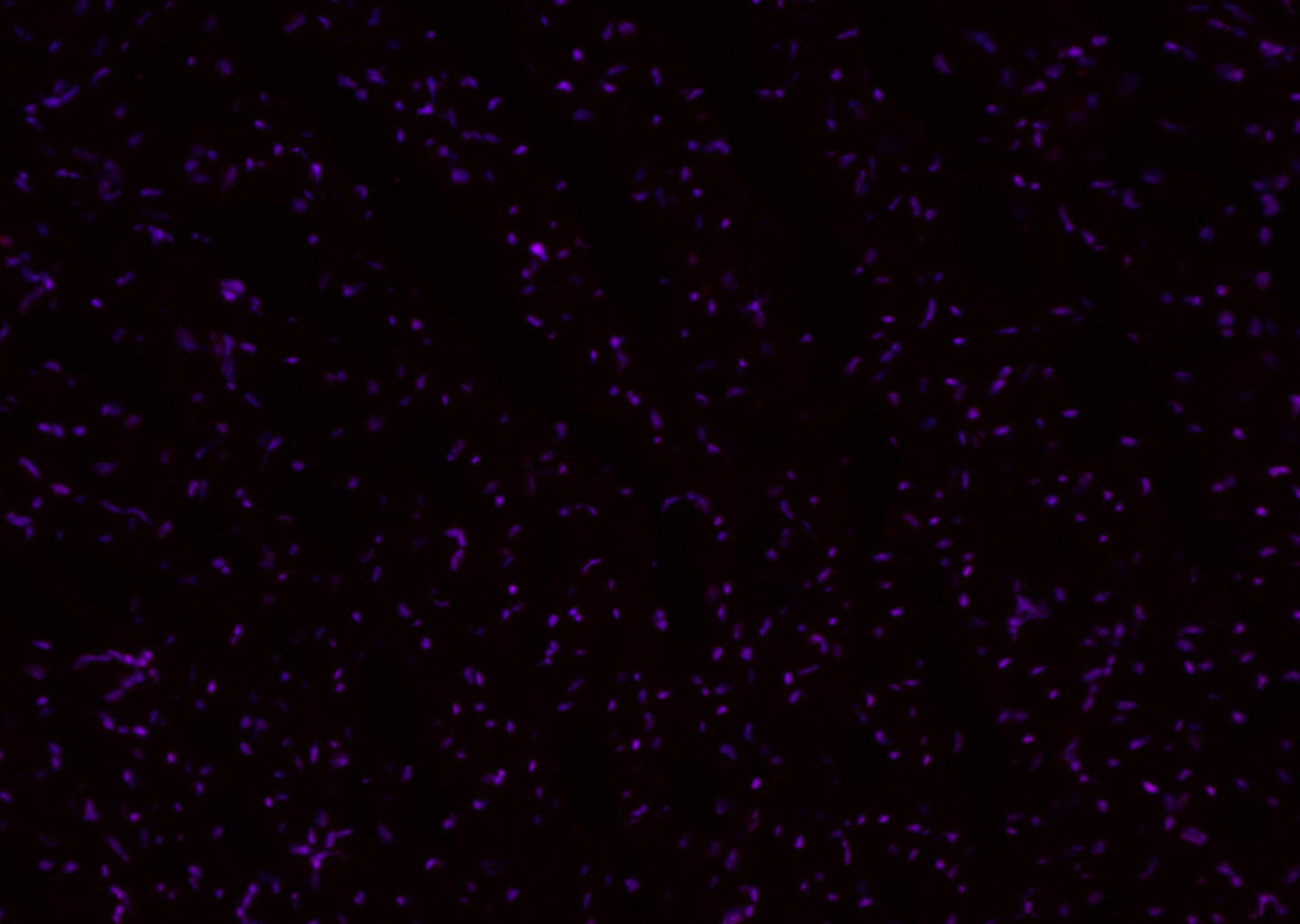

Supplement: Supplementary file 1 [file DataSheet1.ZIP › original source data1/fig2/E/MI-HKE-merge.jpg]

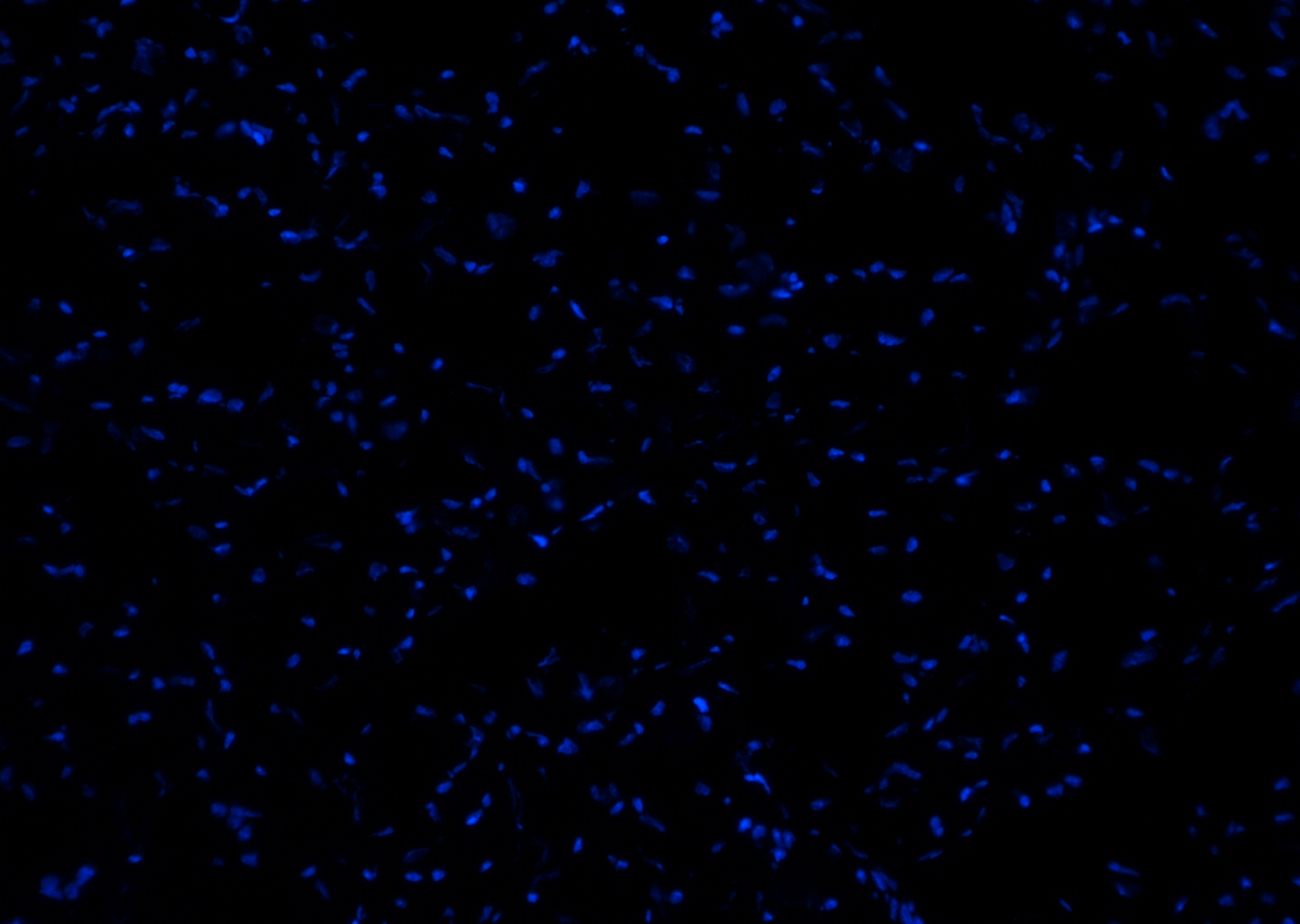

Supplement: Supplementary file 1 [file DataSheet1.ZIP › original source data1/fig2/E/Sham-CT-DAPI.jpg]

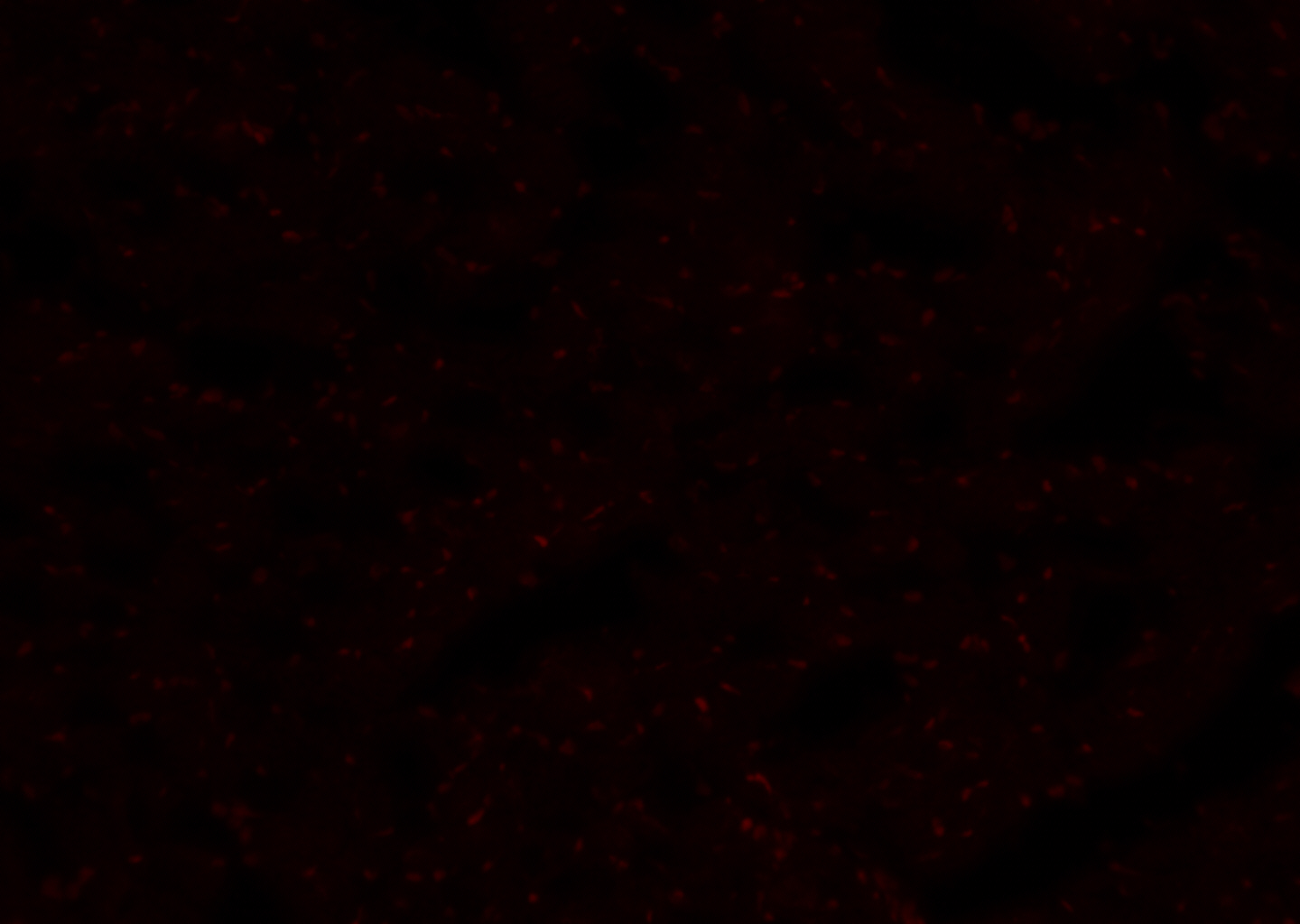

Supplement: Supplementary file 1 [file DataSheet1.ZIP › original source data1/fig2/E/Sham-CT-DHE.jpg]

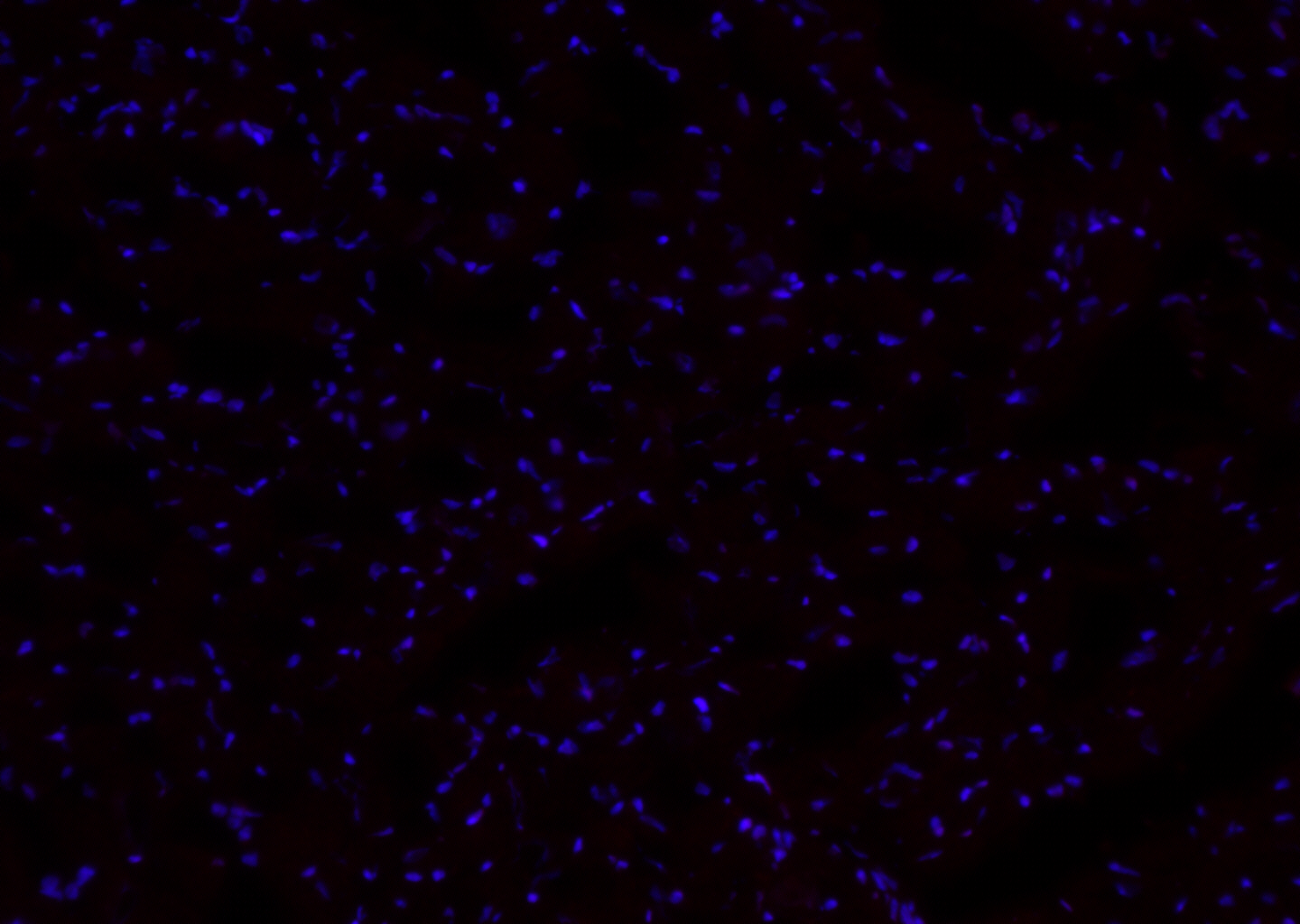

Supplement: Supplementary file 1 [file DataSheet1.ZIP › original source data1/fig2/E/Sham-CT-merge.jpg]

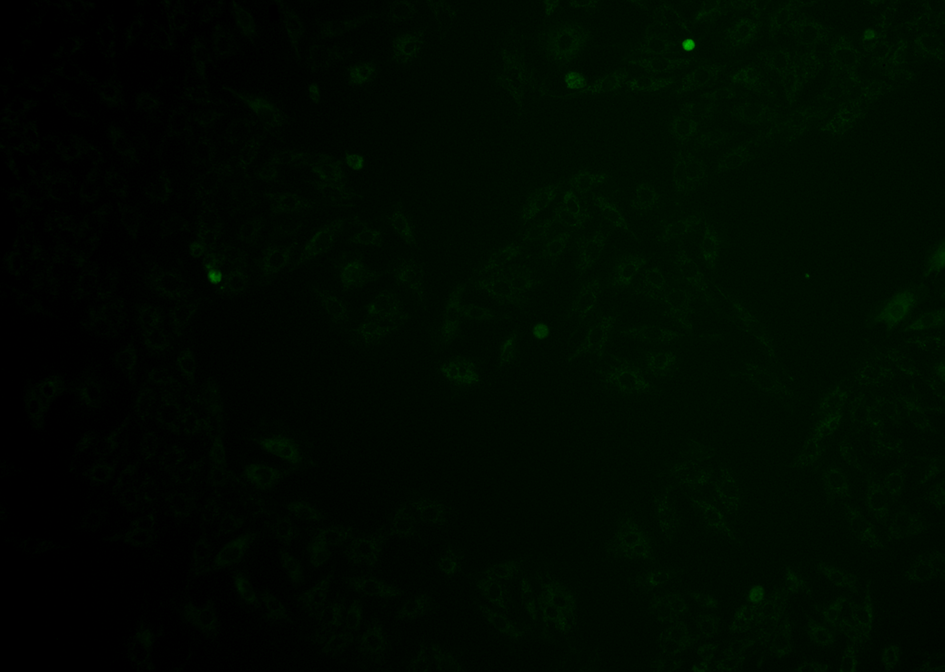

Supplement: Supplementary file 1 [file DataSheet1.ZIP › original source data1/fig3/A/Blank-green.tif]

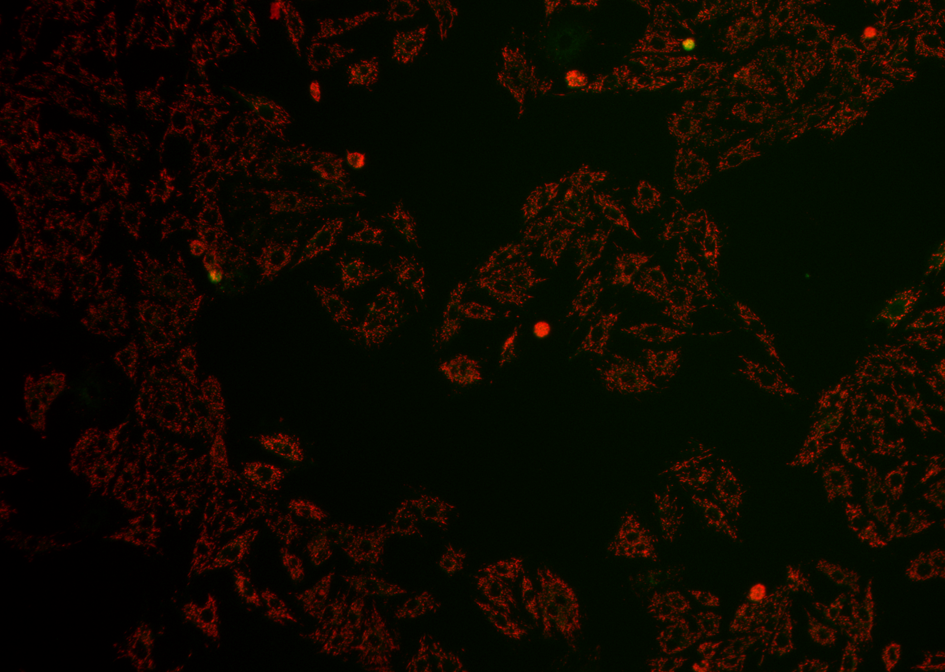

Supplement: Supplementary file 1 [file DataSheet1.ZIP › original source data1/fig3/A/Blank-merge.tif]

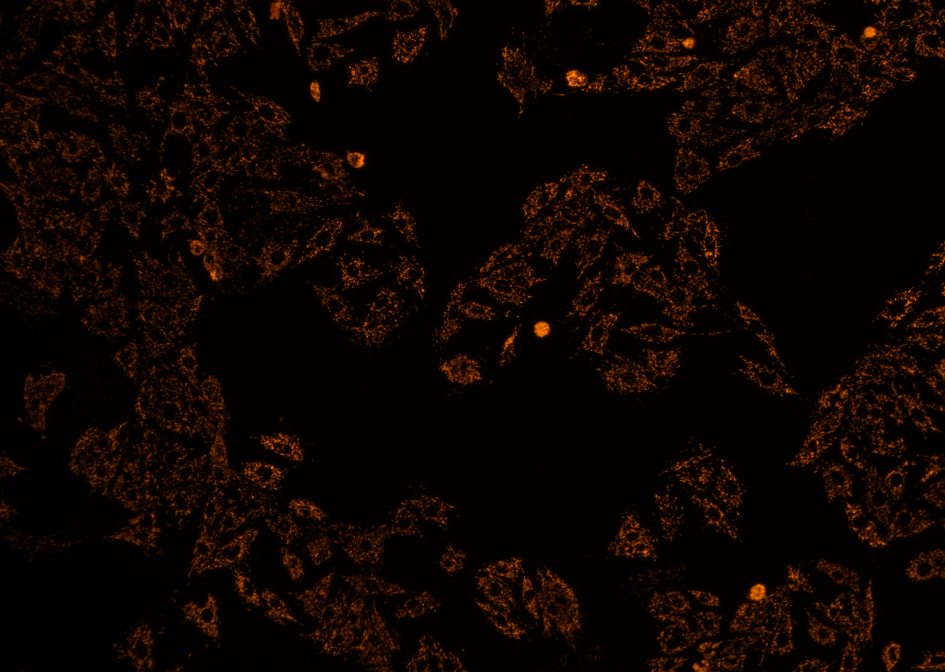

Supplement: Supplementary file 1 [file DataSheet1.ZIP › original source data1/fig3/A/Blank-red.tif]

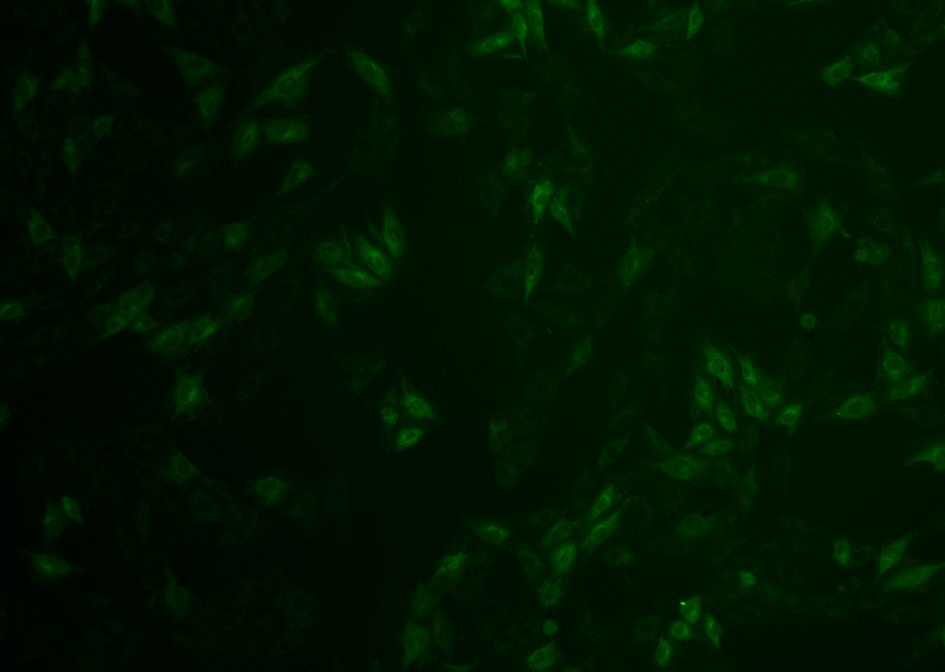

Supplement: Supplementary file 1 [file DataSheet1.ZIP › original source data1/fig3/A/H2O2-green.tif]

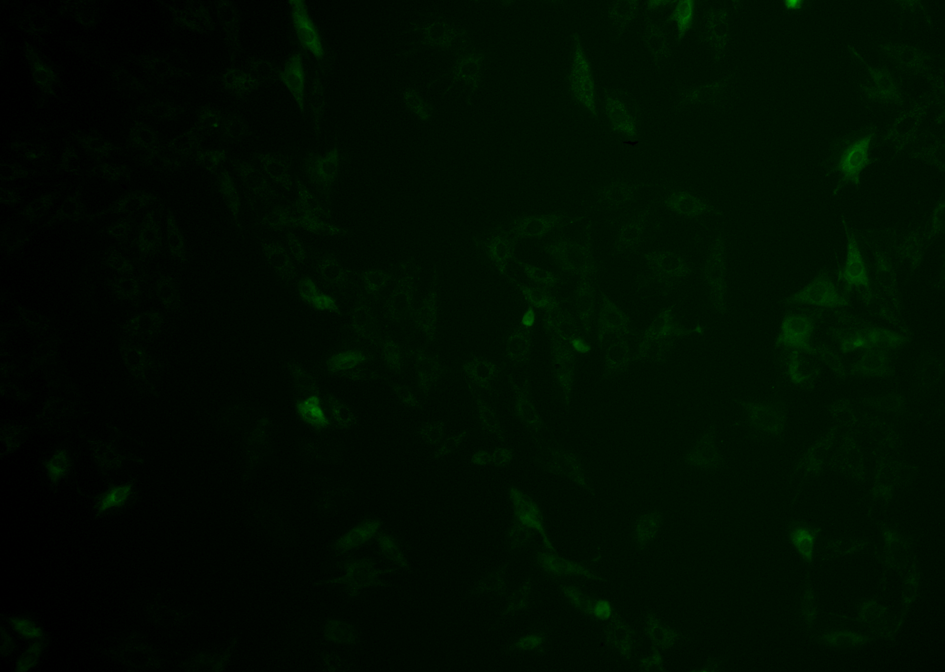

Supplement: Supplementary file 1 [file DataSheet1.ZIP › original source data1/fig3/A/H2O2-HK-green.tif]

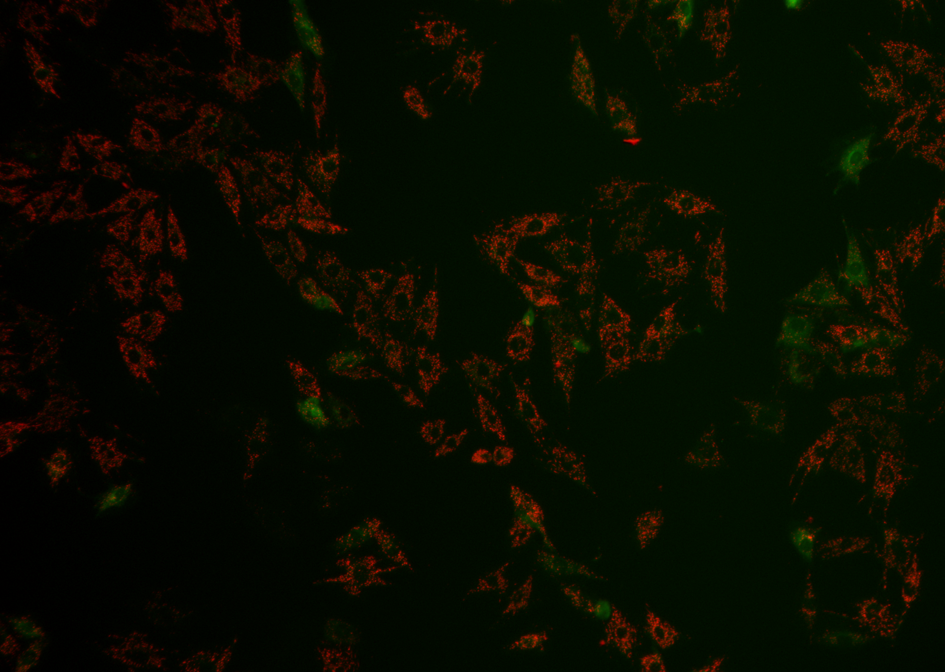

Supplement: Supplementary file 1 [file DataSheet1.ZIP › original source data1/fig3/A/H2O2-HK-merge.tif]

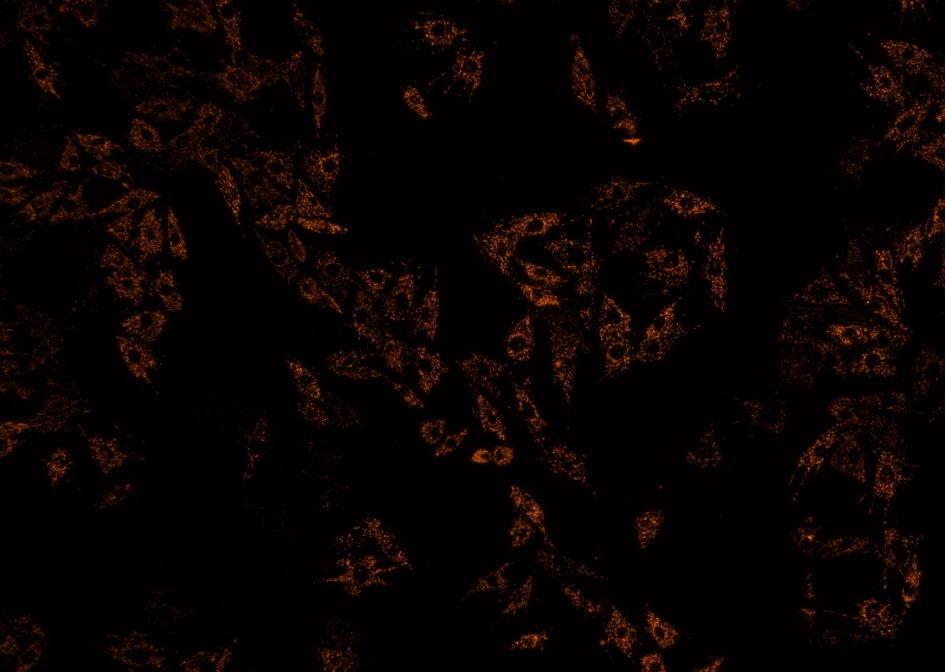

Supplement: Supplementary file 1 [file DataSheet1.ZIP › original source data1/fig3/A/H2O2-HK-red.tif]

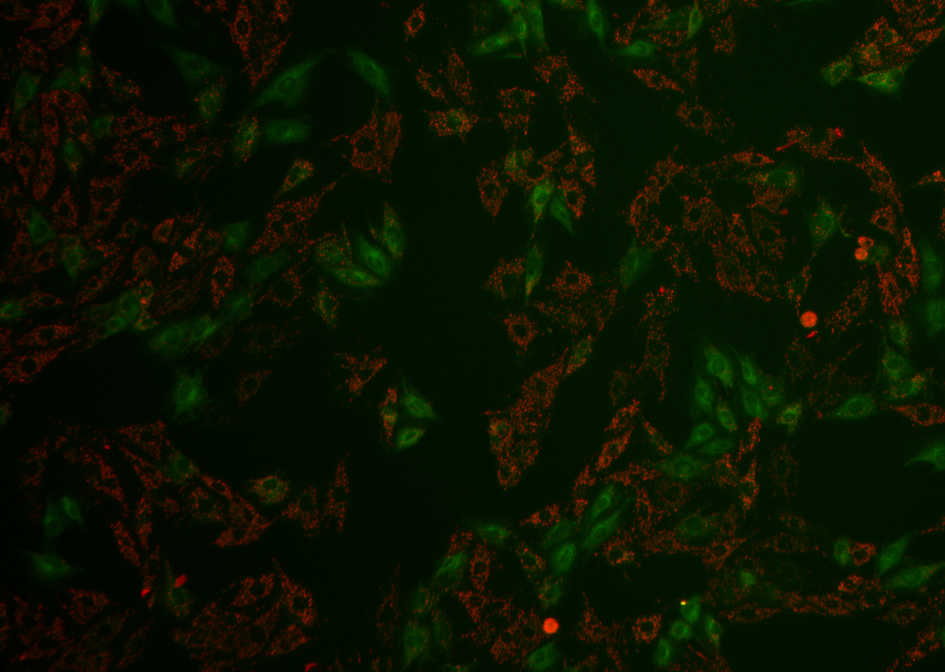

Supplement: Supplementary file 1 [file DataSheet1.ZIP › original source data1/fig3/A/H2O2-merge.tif]

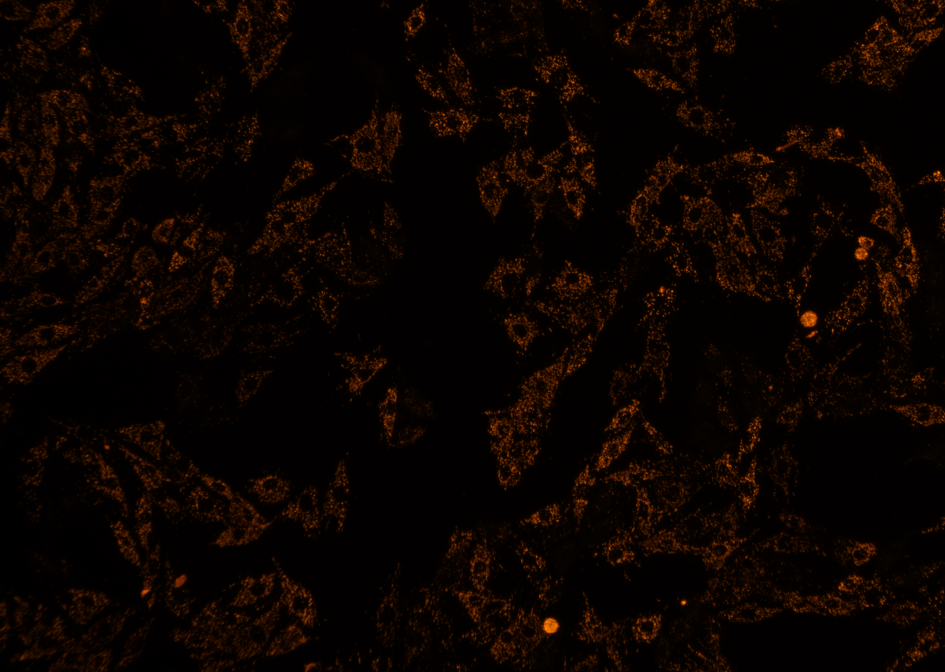

Supplement: Supplementary file 1 [file DataSheet1.ZIP › original source data1/fig3/A/H2O2-red.tif]

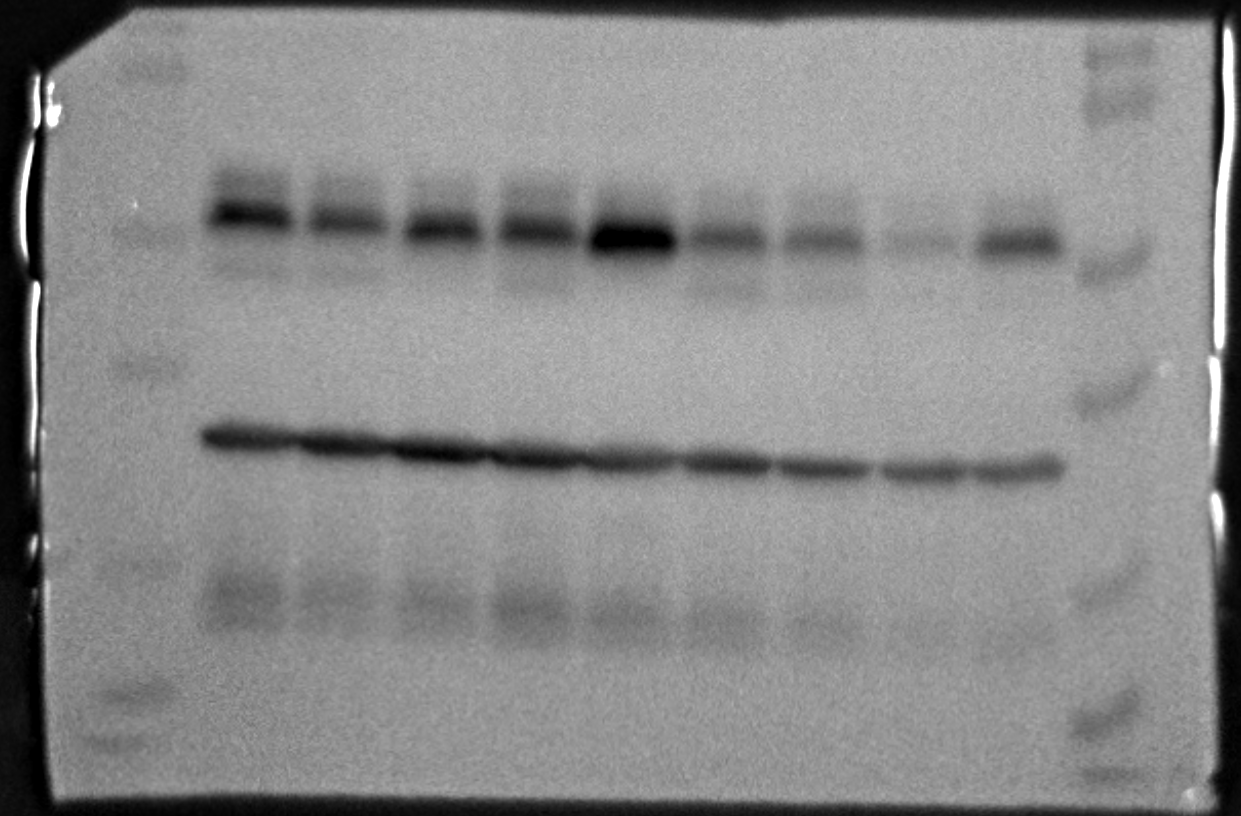

Supplement: Supplementary file 1 [file DataSheet1.ZIP › original source data1/fig5/D/Fig.5-Gapdh.tif]

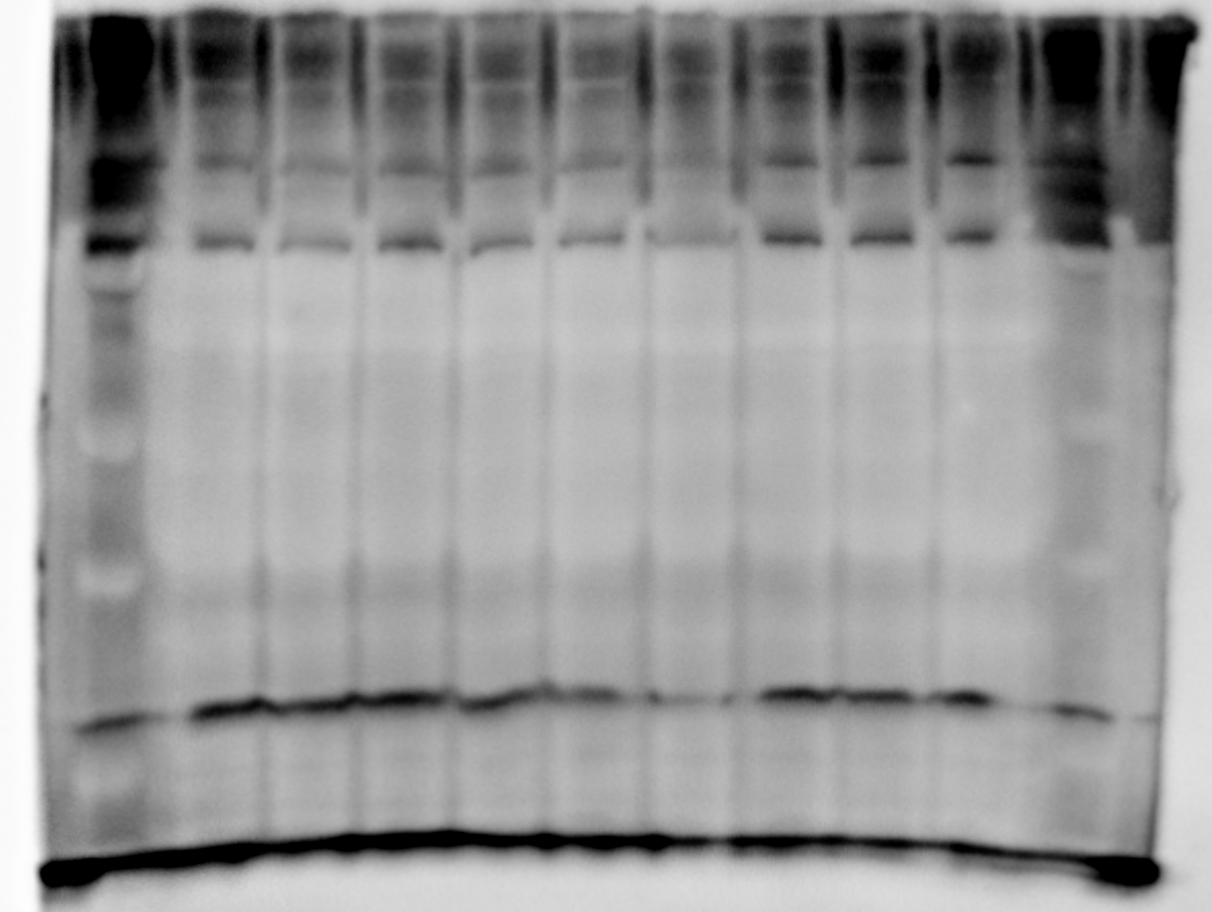

Supplement: Supplementary file 1 [file DataSheet1.ZIP › original source data1/fig5/D/Fig.5-Ucp3.tif]

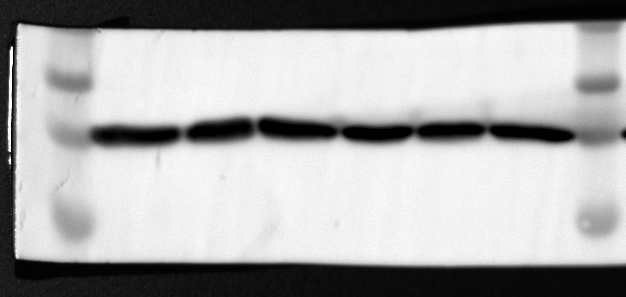

Supplement: Supplementary file 1 [file DataSheet1.ZIP › original source data1/fig5/F/Fig.5-293GAPDH.tif]

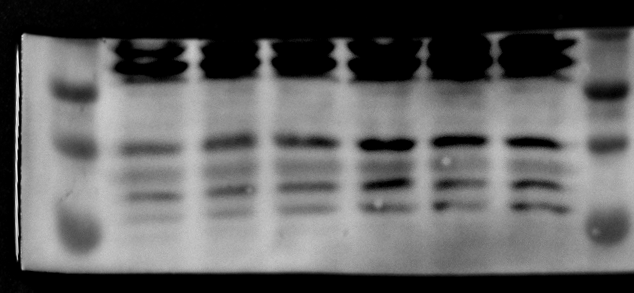

Supplement: Supplementary file 1 [file DataSheet1.ZIP › original source data1/fig5/F/Fig.5-293UCP3.tif]

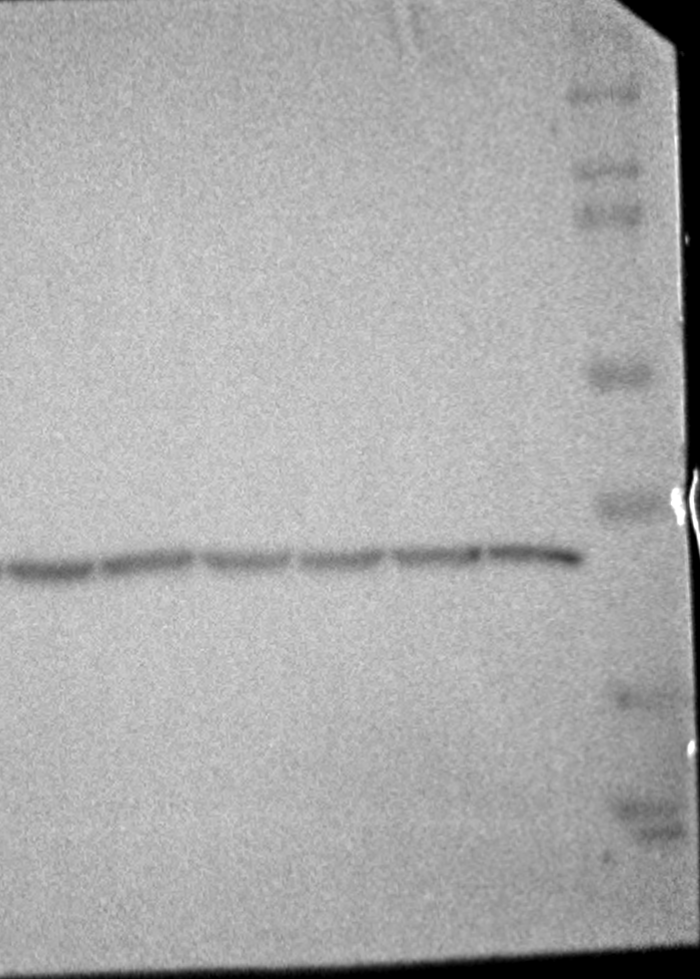

Supplement: Supplementary file 1 [file DataSheet1.ZIP › original source data1/fig5/F/Fig.5-H9c2Gapdh.tif]

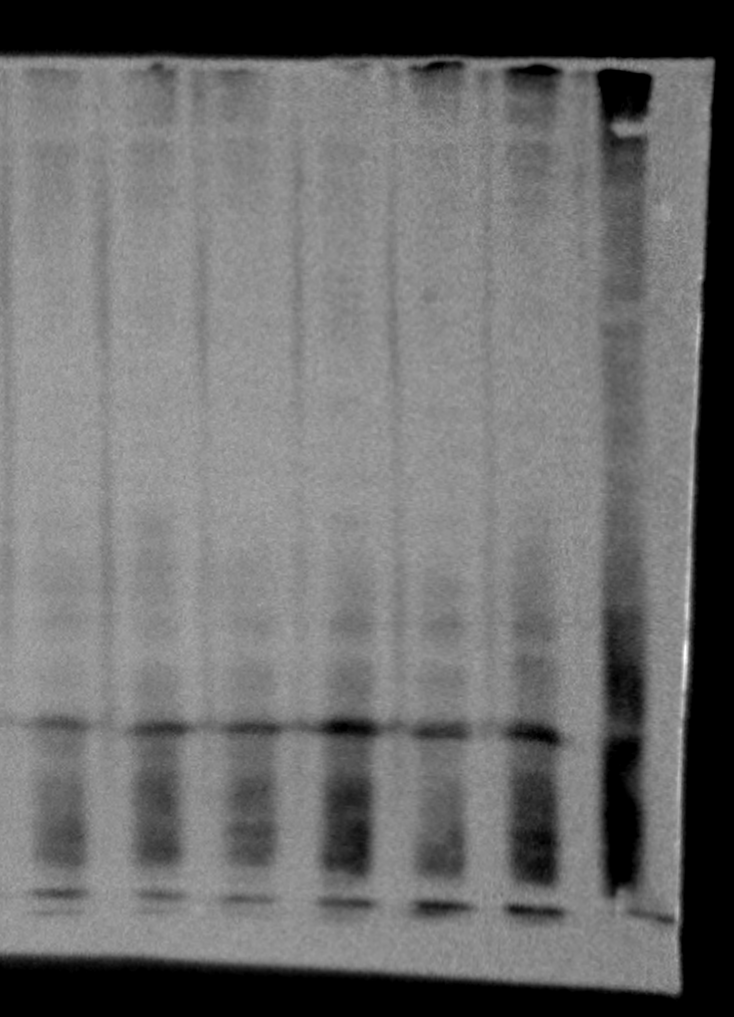

Supplement: Supplementary file 1 [file DataSheet1.ZIP › original source data1/fig5/F/Fig.5-H9c2Ucp3.tif]

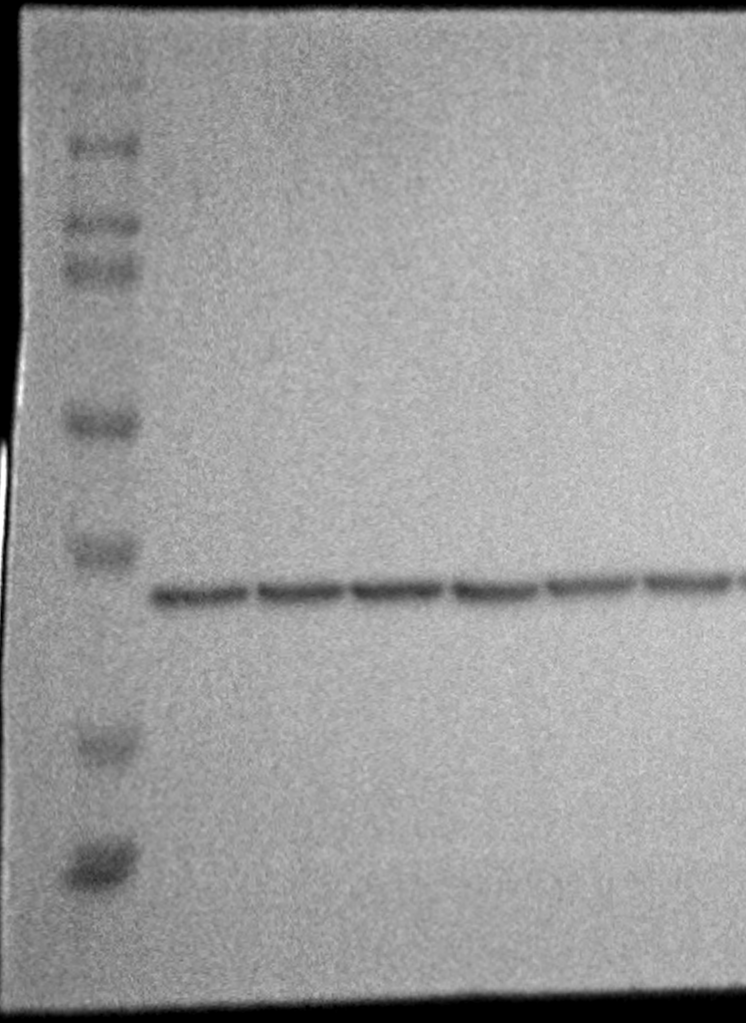

Supplement: Supplementary file 1 [file DataSheet1.ZIP › original source data1/fig5/F/Fig.5-PCGapdh.tif]

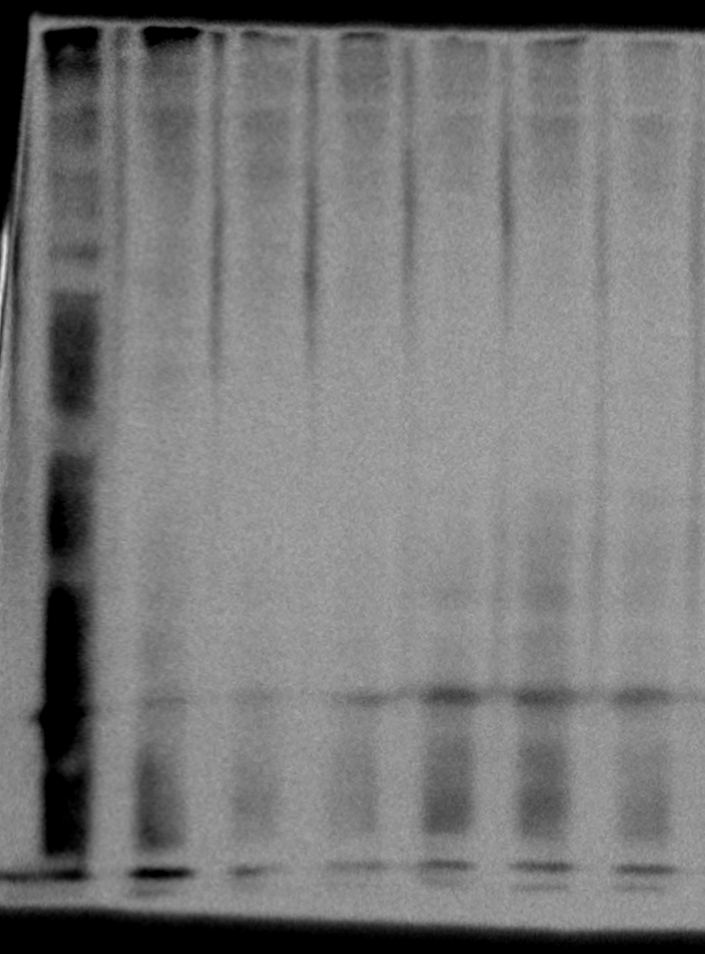

Supplement: Supplementary file 1 [file DataSheet1.ZIP › original source data1/fig5/F/Fig.5-PCUcp3.tif]

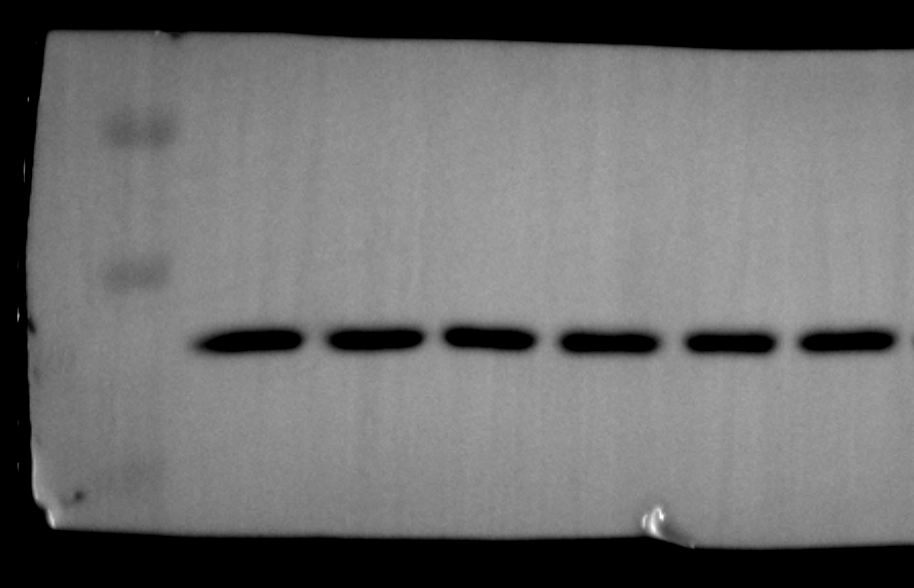

Supplement: Supplementary file 2 [file DataSheet2.ZIP › original source data2/fig.s8/Fig.S6-Gapdh.tif]

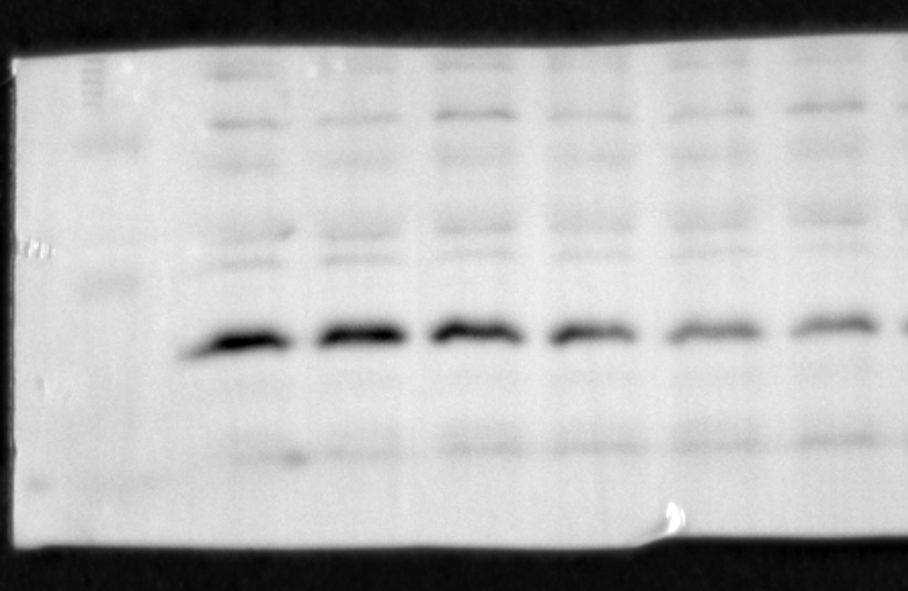

Supplement: Supplementary file 2 [file DataSheet2.ZIP › original source data2/fig.s8/Fig.S6-Ucp3.tif]

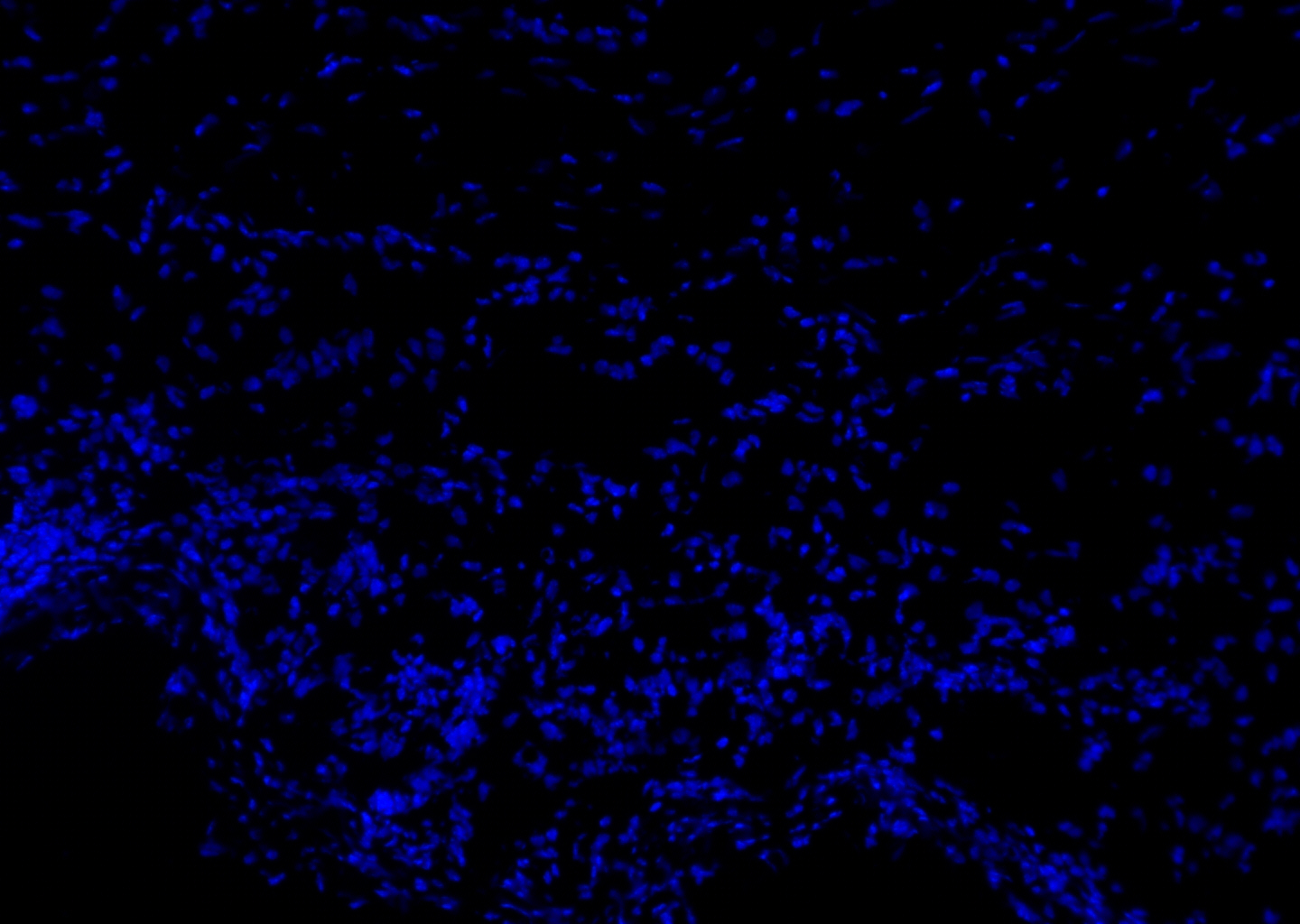

Supplement: Supplementary file 2 [file DataSheet2.ZIP › original source data2/fig7/D/HKE-KO-DAPI.jpg]

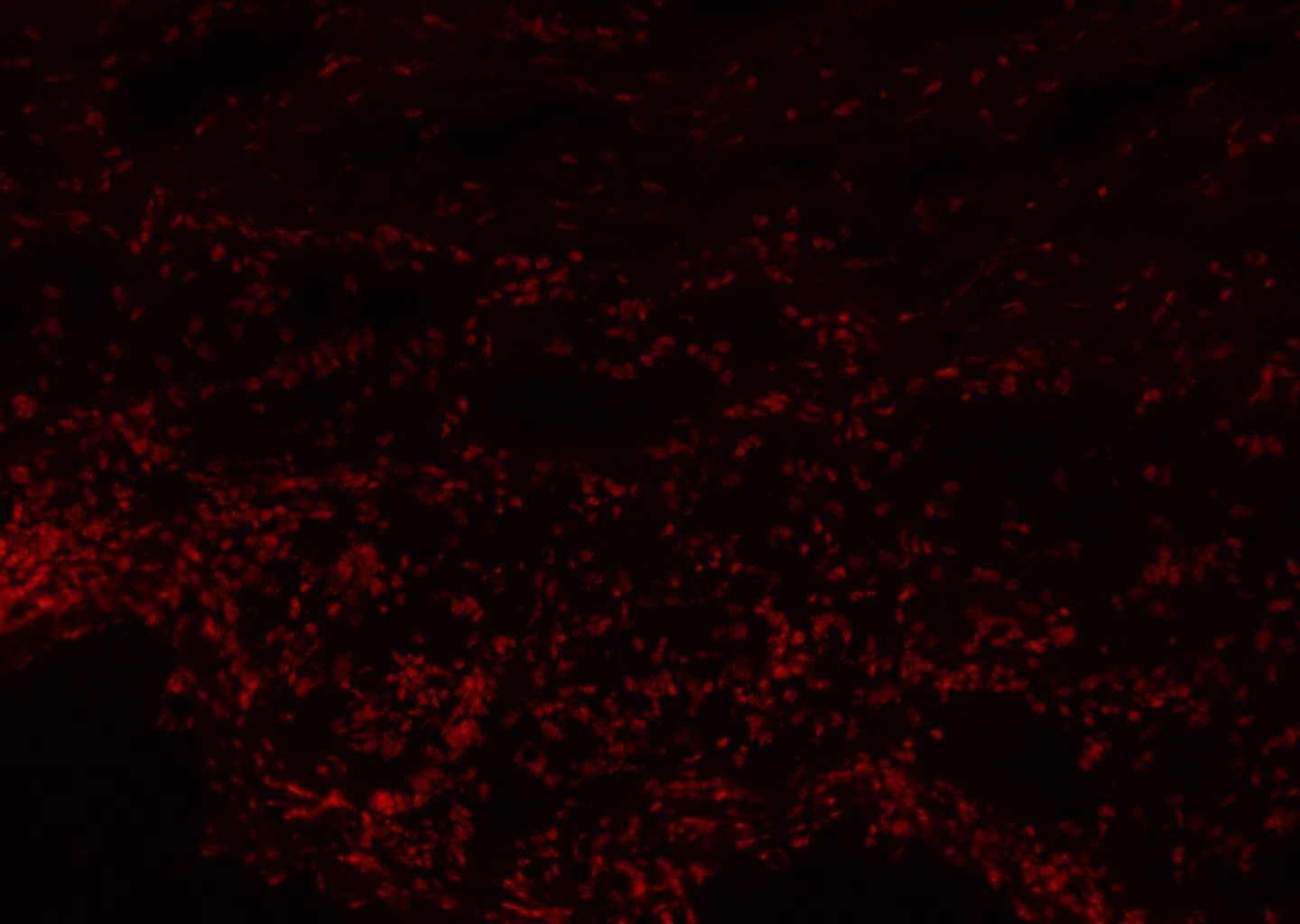

Supplement: Supplementary file 2 [file DataSheet2.ZIP › original source data2/fig7/D/HKE-KO-DHE.jpg]

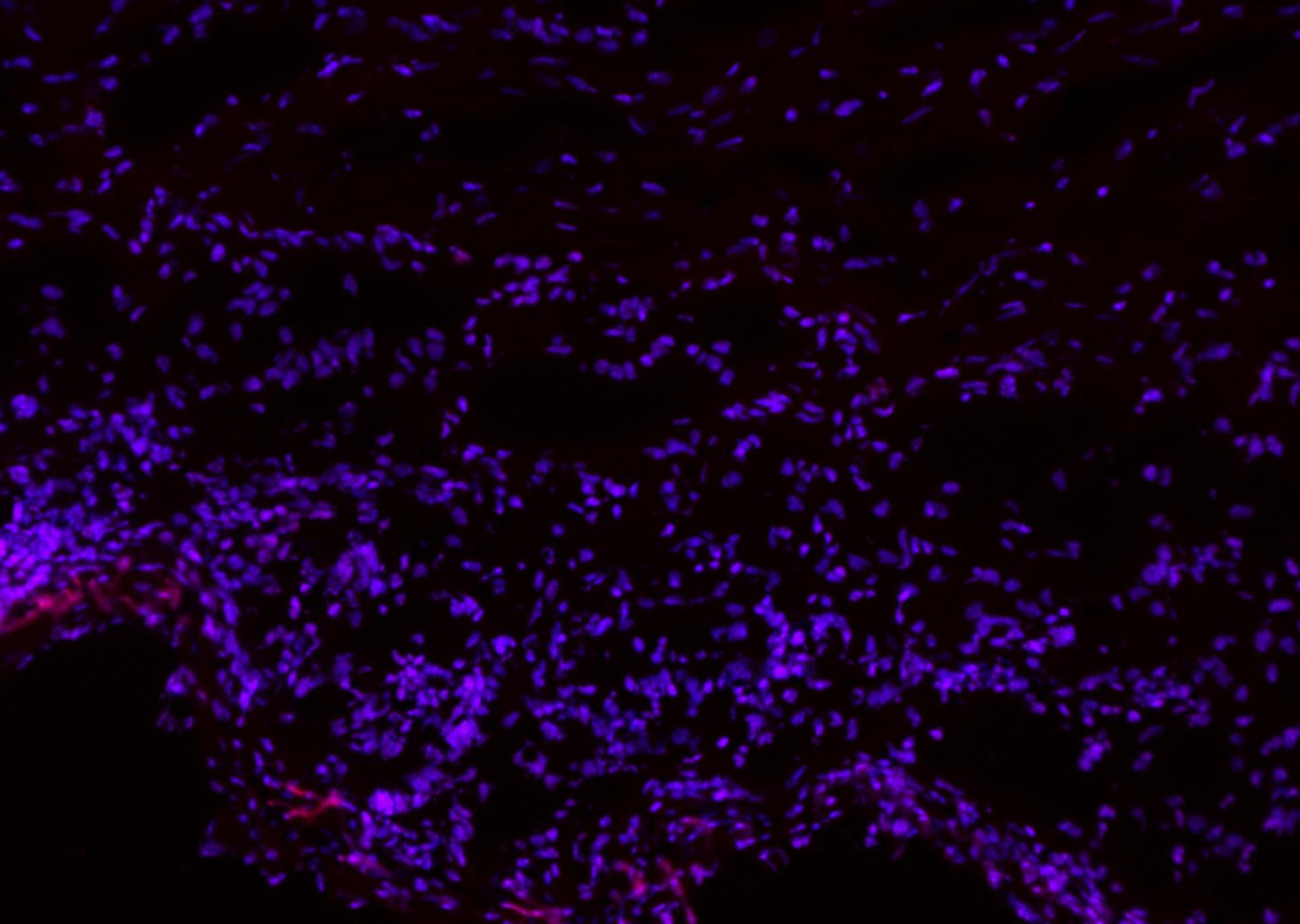

Supplement: Supplementary file 2 [file DataSheet2.ZIP › original source data2/fig7/D/HKE-KO-MERGE.jpg]

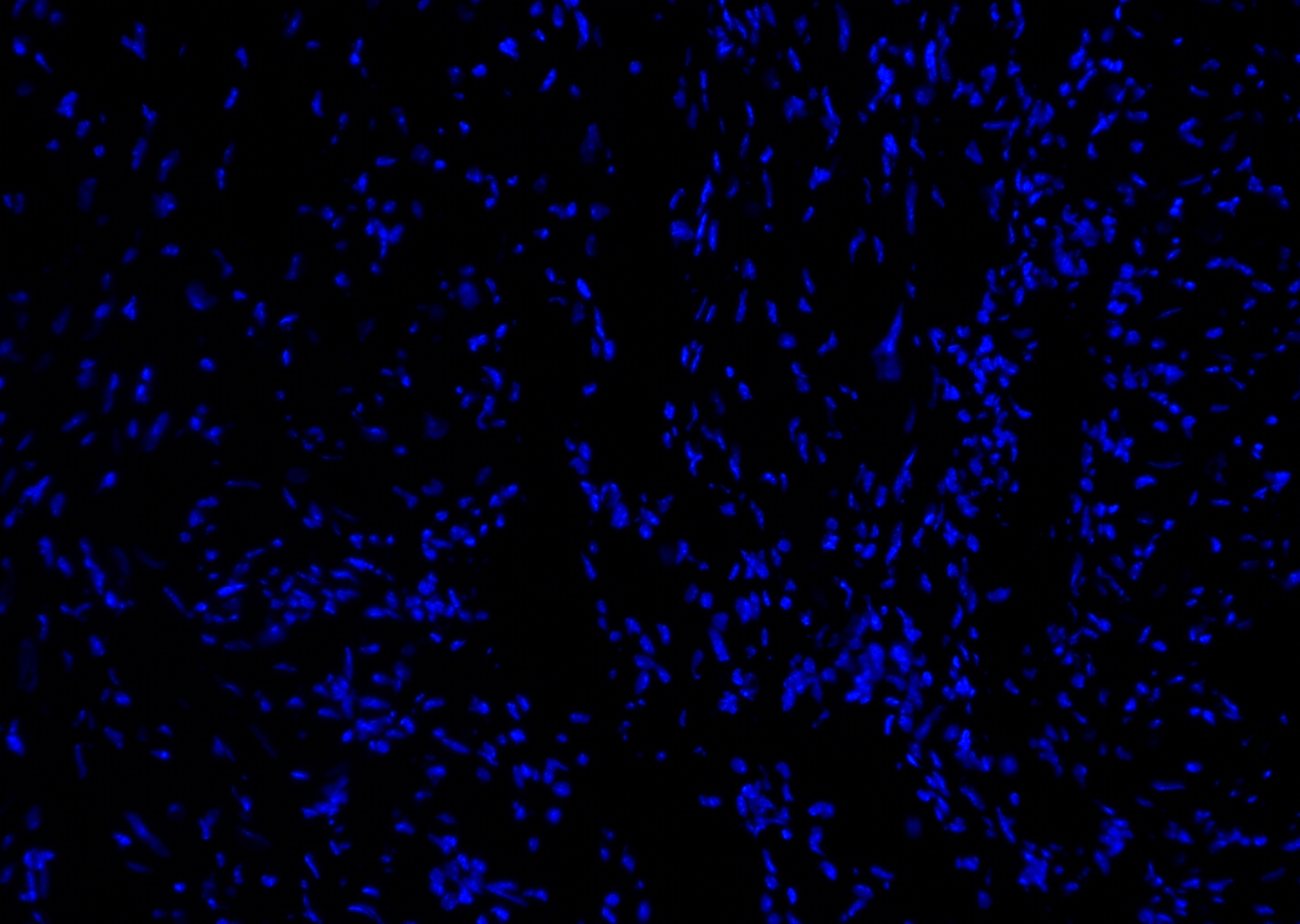

Supplement: Supplementary file 2 [file DataSheet2.ZIP › original source data2/fig7/D/HKE-WT-DAPI.jpg]

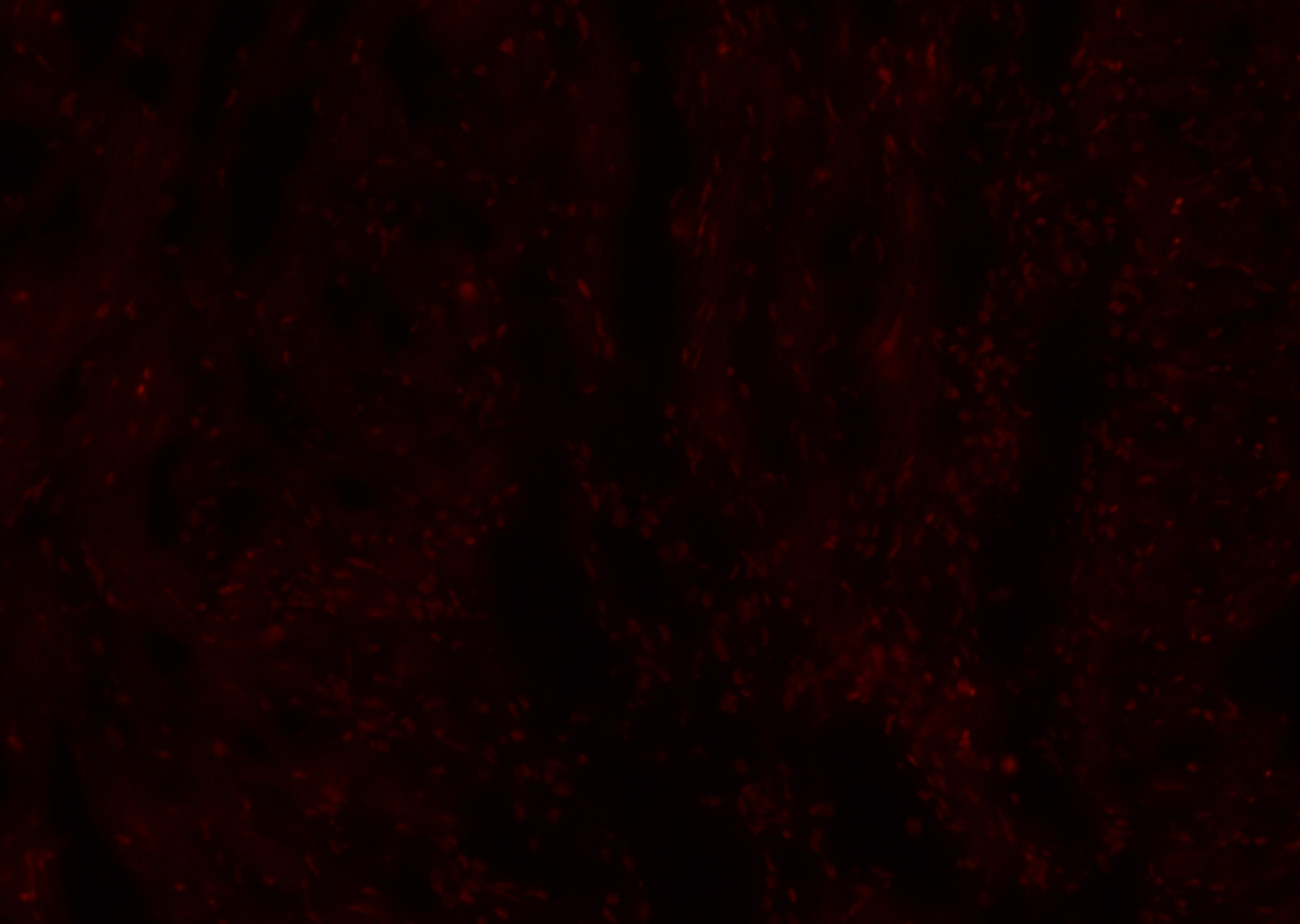

Supplement: Supplementary file 2 [file DataSheet2.ZIP › original source data2/fig7/D/HKE-WT-DHE.jpg]

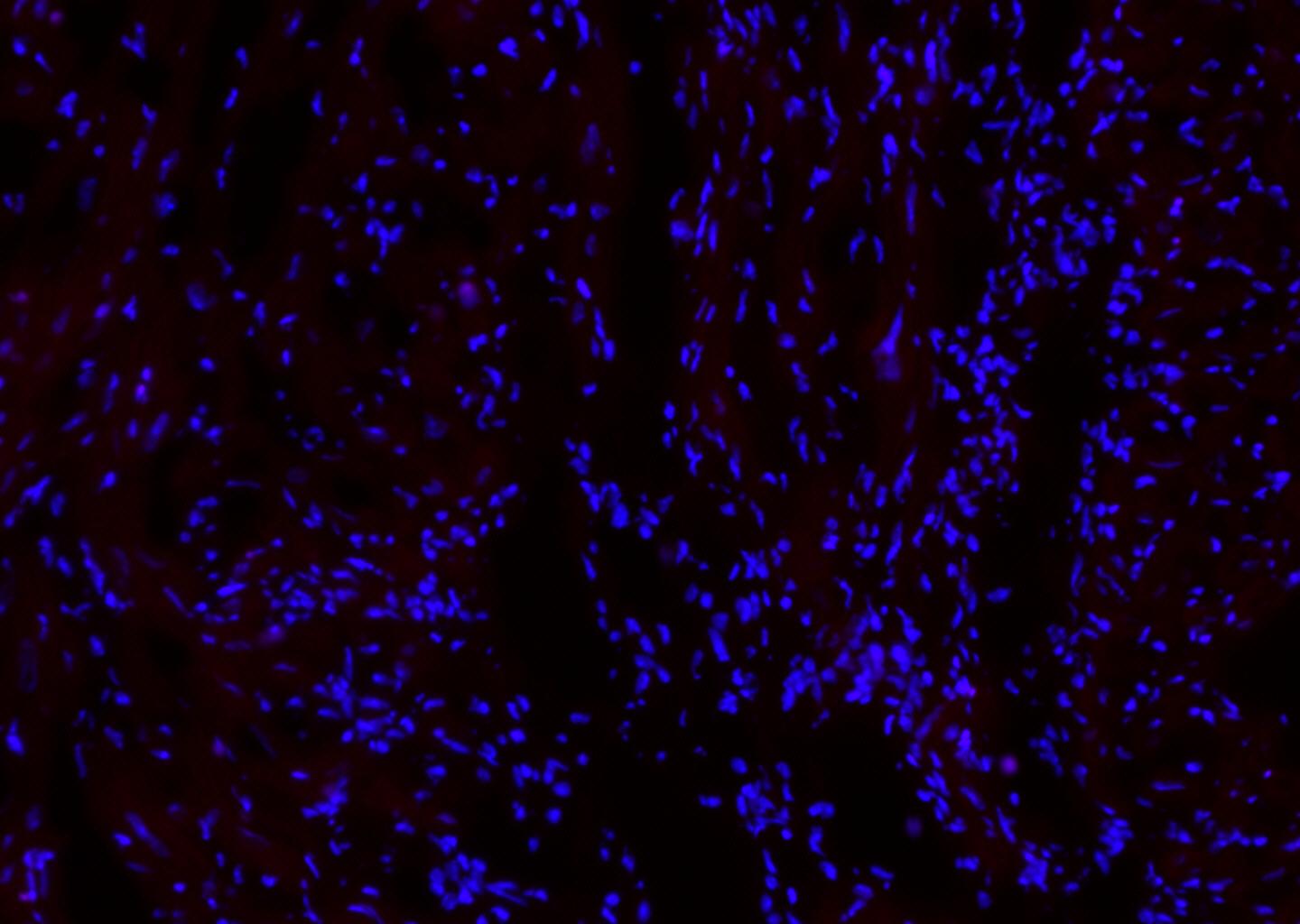

Supplement: Supplementary file 2 [file DataSheet2.ZIP › original source data2/fig7/D/HKE-WT-MERGE.jpg]

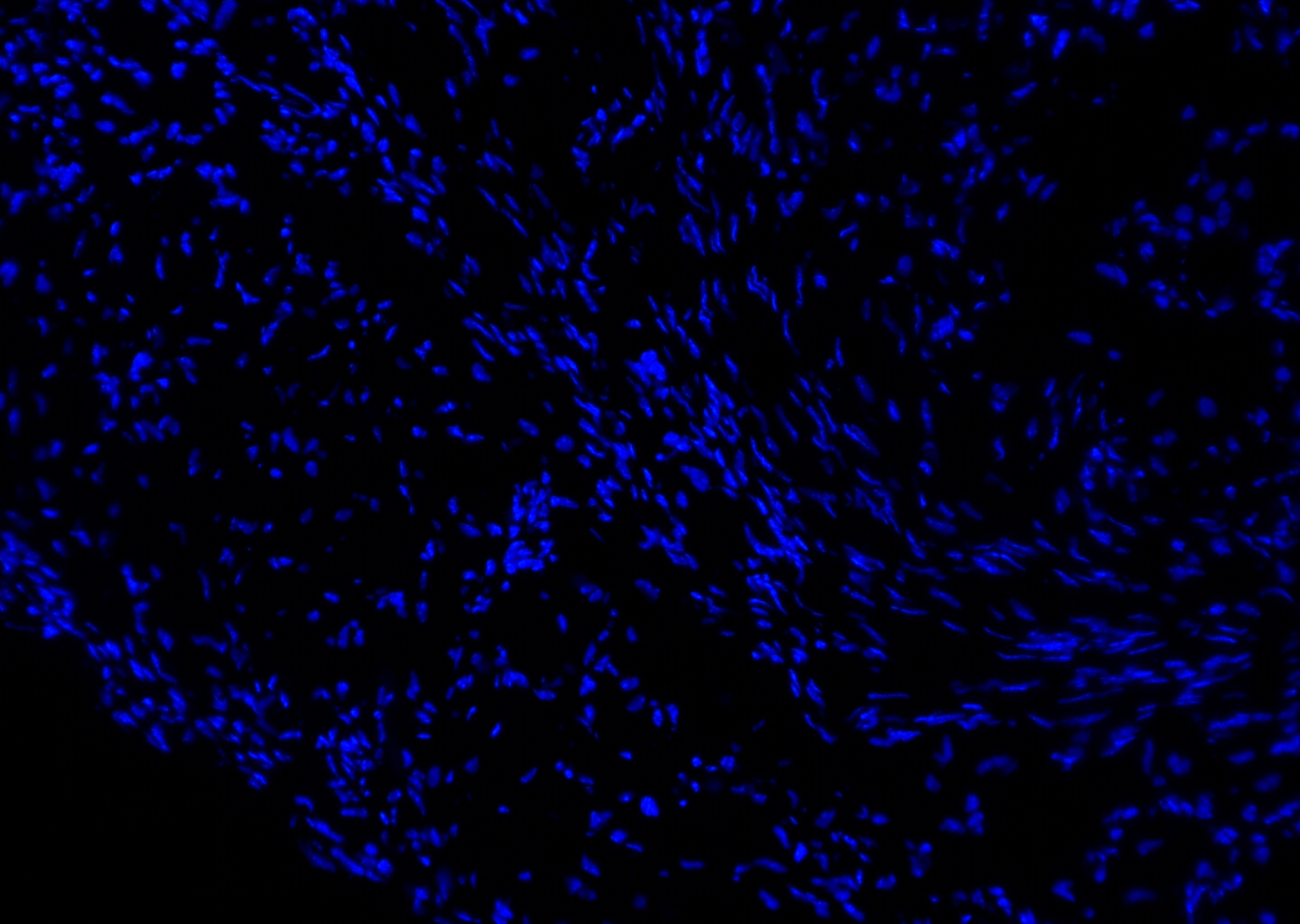

Supplement: Supplementary file 2 [file DataSheet2.ZIP › original source data2/fig7/D/MI-DAPI.jpg]

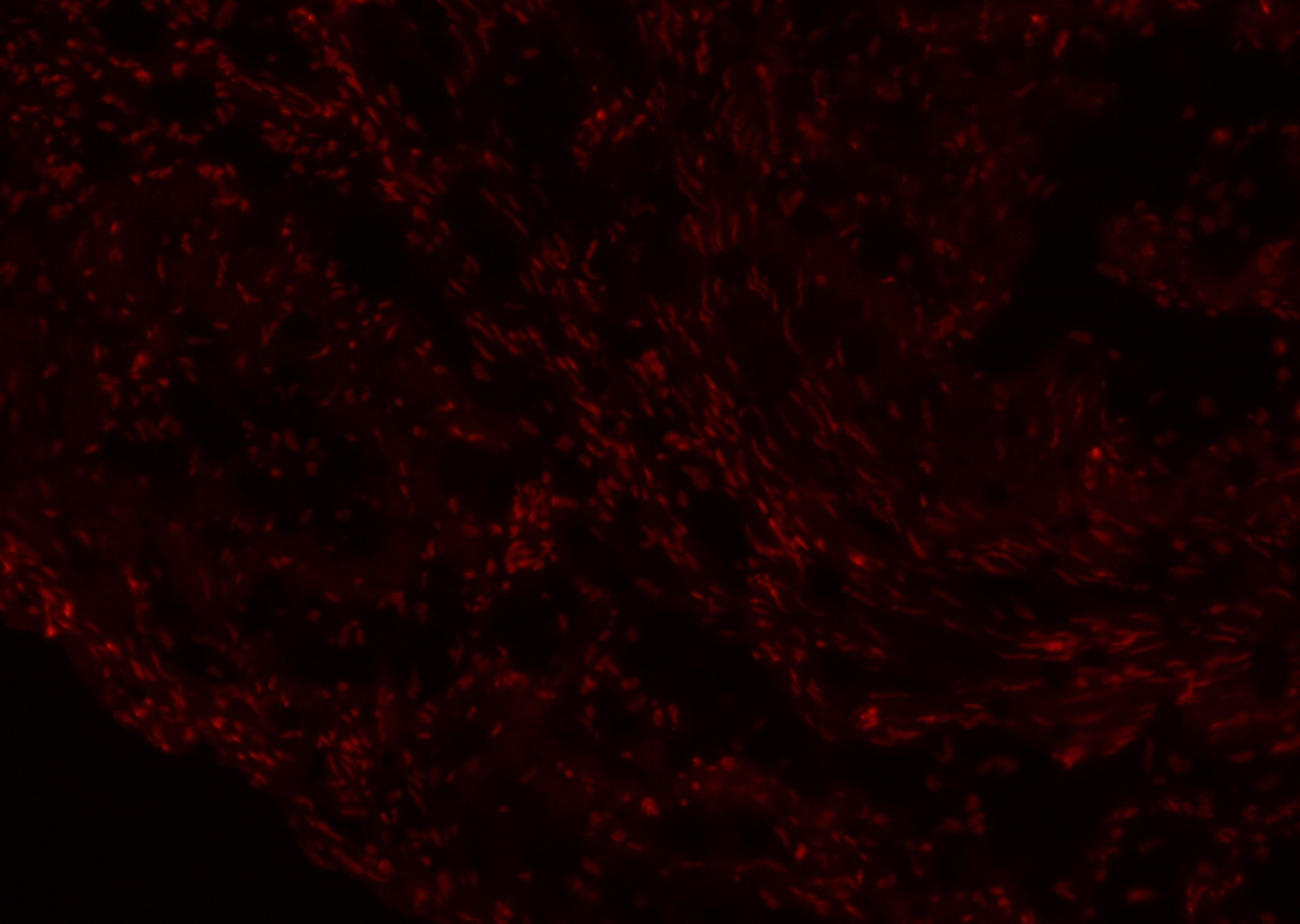

Supplement: Supplementary file 2 [file DataSheet2.ZIP › original source data2/fig7/D/MI-DHE.jpg]

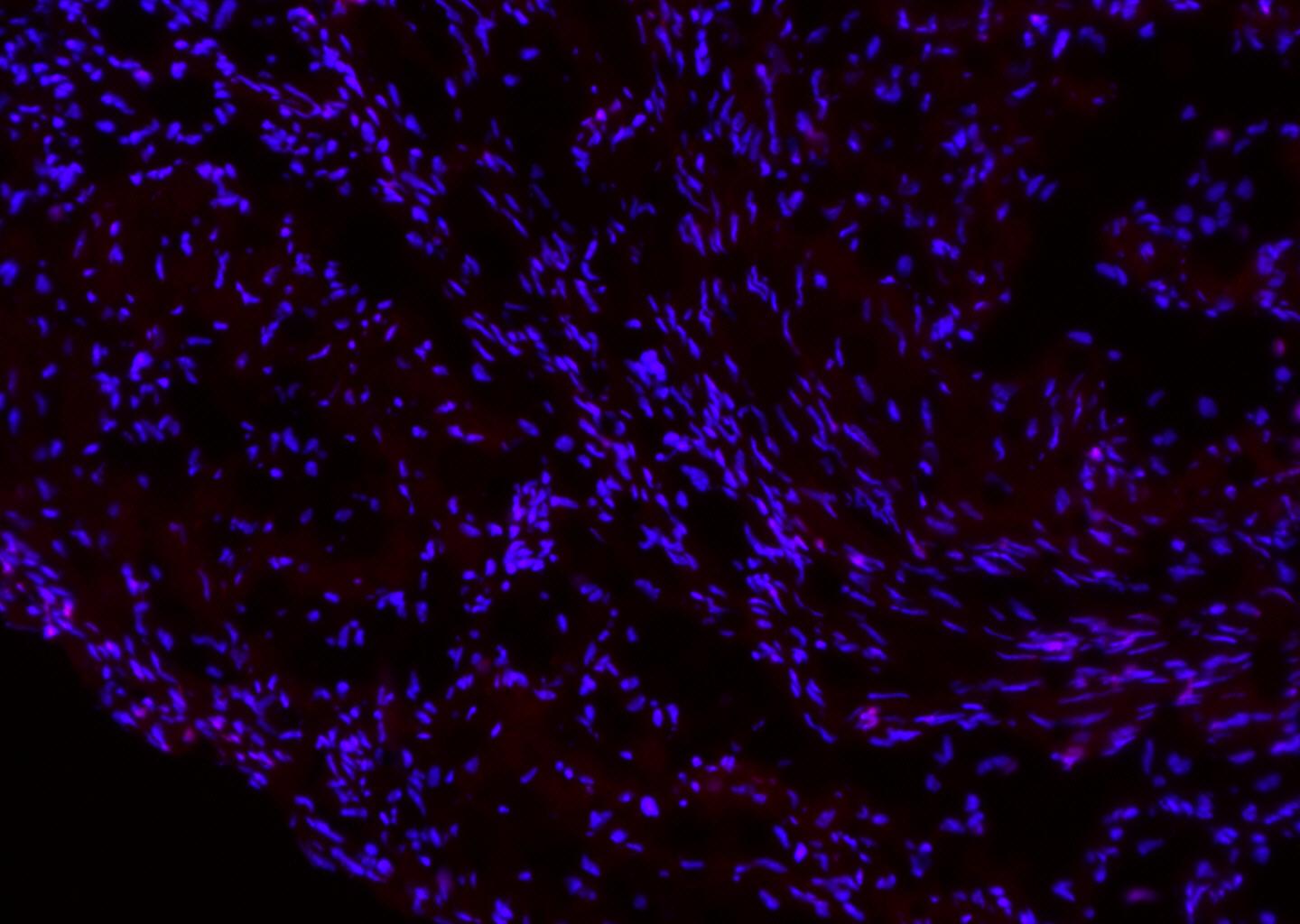

Supplement: Supplementary file 2 [file DataSheet2.ZIP › original source data2/fig7/D/MI-MERGE.jpg]
