# Supplementary material for: Honokiol Ameliorates Post-Myocardial Infarction Heart Failure Through Ucp3-Mediated Reactive Oxygen Species Inhibition
Source: Front Pharmacol. 2022 Feb 21;13:811682. doi: 10.3389/fphar.2022.811682 (PMC8899544; doi:10.3389/fphar.2022.811682)
Supplement: Supplementary file 3 [file DataSheet1.docx]

Supplementary Material





**Supplementary Figure 1.** Survival rates in groups Sham/CT (10), MI/CT (15), and MI/HKE (15).


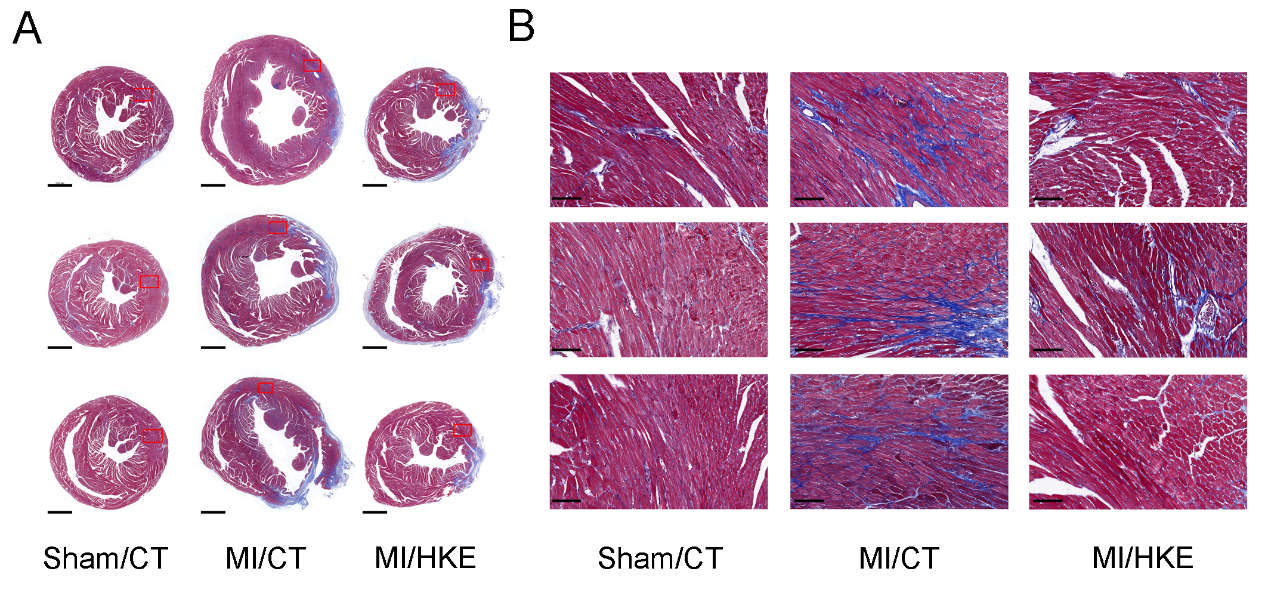


**Supplementary Figure 2.** Masson’s Trichrome staining was conducted to evaluate myocardial fibrosis, the fibrotic area was indicated as a blue region, the red frame encircles the fibrosis away from the infarct area in the MI group and similar locations in other groups, scale bar: 1mm (A). Enlarged view of red frame, scale bar:50μm (B).





**Supplementary Figure 3.** To determine the concentration of HK, H9c2 cells were seeded in 96 well plates for 12 h in the presence of 10/20/40/60/80μM HK or DMSO, the medium was then replaced with normal medium containing 400μM hydrogen peroxide for 12 h to induce oxidative damage. Then, 15 μL MTT (0.5 mg/mL) was added to each well, and the plates were incubated for 2 h. After that, the medium was replaced with 200 μL of DMSO, and the absorbance was recorded at 570 nm, cell viability is the ratio of the OD value of each treatment to the untreated group. Each experiment was repeated three times. **P < 0.05*, ***P < 0.01* vs control group, *^#^P* < 0.05, *^##^P* < 0.01 vs 400μM H_2_O_2_ treatment group.


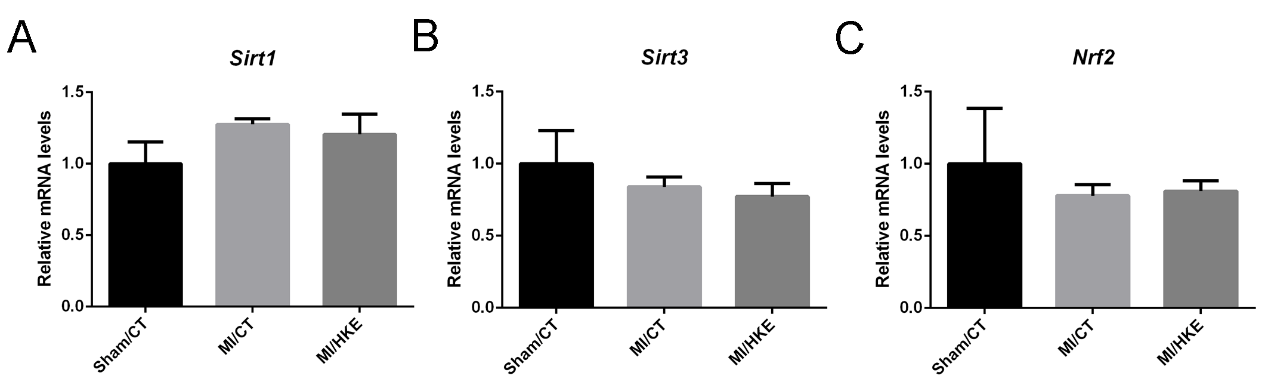


**Supplementary Figure 4.** The expression of *Sirt1*, *Sirt3* and *Nrf2*. The mRNA expression of *Sirt1*, *Sirt3* and *Nrf2* in myocardial tissue was detected using qRT-PCR (n = 3 per group). 2^-△△Ct^ method was used to analyze relative gene expression levels(A-C). Values are shown as means ± SEM, **P < 0.05*, ***P < 0.01* vs Sham/CT group, #*P < 0.05*, ##*P < 0.01* vs MI/CT group.


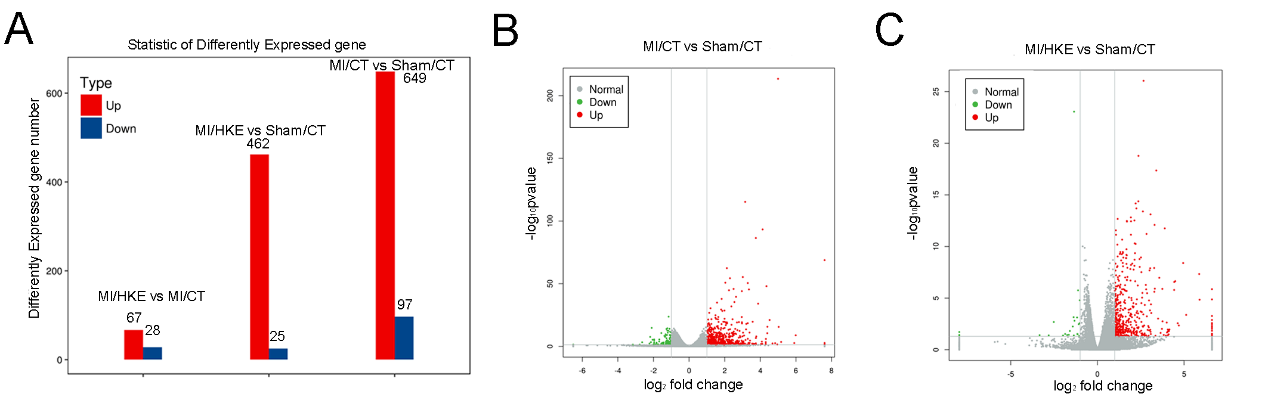


**Supplementary Figure 5.** Differentially expressed gene statistics. The data comes from the sample expression value in the sequencing results, the calculation of pvalue: negative binomial distribution test H0: pvalue of A = B (n = 3 per group).


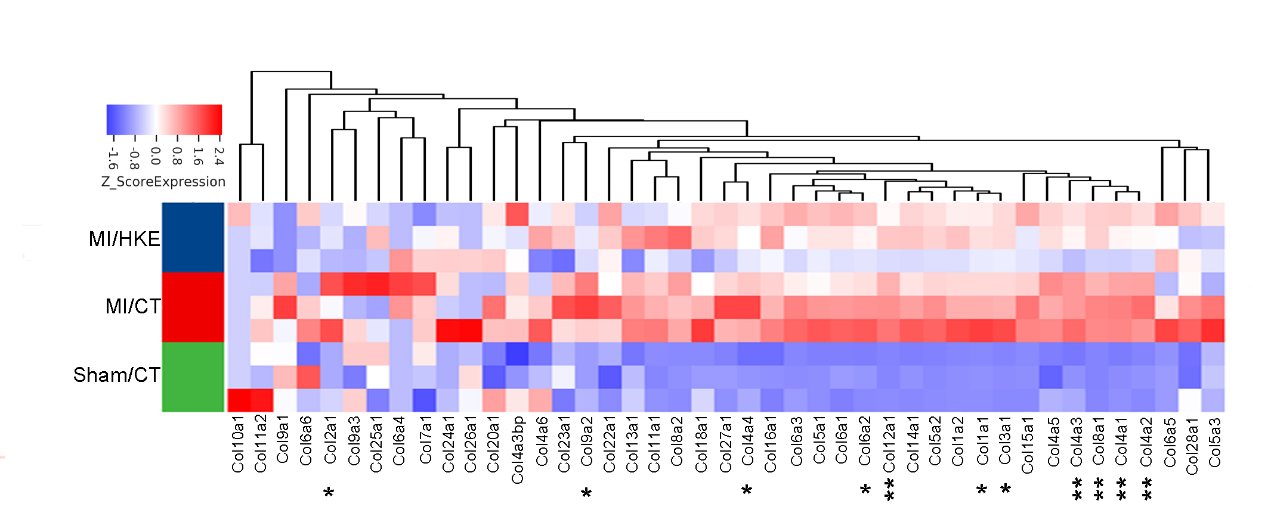


**Supplementary Figure 6.** Cluster analysis of 43 collagen-coding genes. In the MI model, the collagen coding genes are affected by HK, and 11 of them are significantly different.





**Supplementary Figure 7.** HK upregulates Ucp3 expression in cardiomyocytes. After 40μM HK treatment, the mRNA expression of Ucp3 in neonatal mice cardiomyocytes was detected using Real-time PCR (n = 3 per group). 2^-△△Ct^ method was used to analyze relative gene expression levels, Gapdh was included as a loading control. Values are shown as means ± SEM, **P < 0.05* vs 0h.


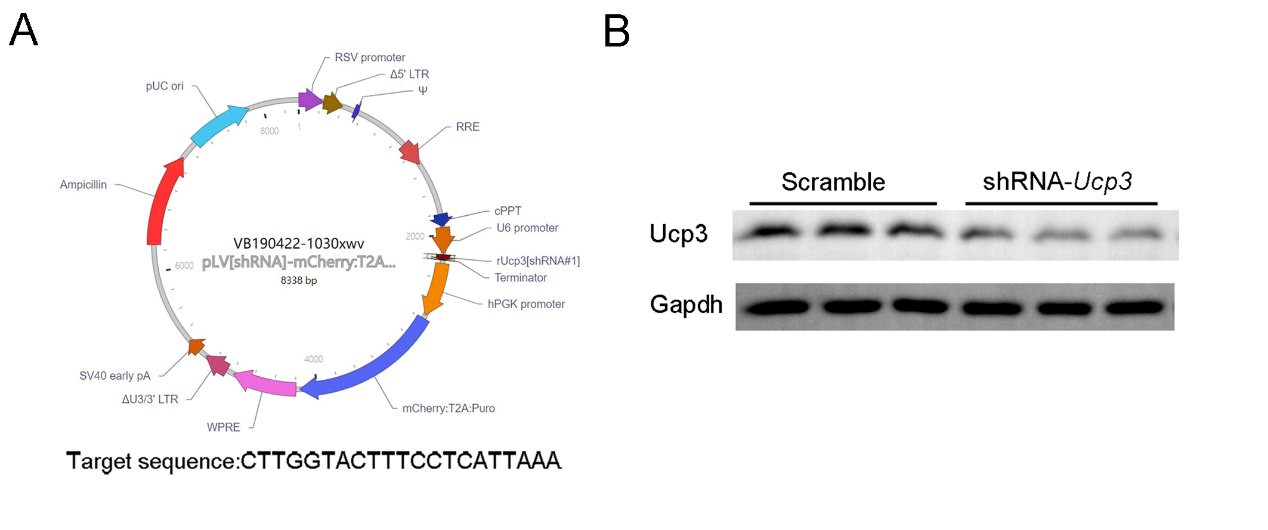


**Supplementary Figure 8.** UCP3 knockdown cell line construction. UCP3 knockdown plasmid structure (A). UCP3 expression of H9c2-based Scramble and shRNA-Ucp3 cells(B).

| **Gene name** | **Sequence** | |
| --- | --- | --- |
| *Sirt1* | F:5’-TGCCATCATGAAGCCAGAGA-3’ | R:5’-AACATCGCAGTCTCCAAGGA-3’ |
| *Sirt3* | F:5’-ATCCCGGACTTCAGATCCCC-3’ | R:5’-CAACATGAAAAAGGGCTTGGG-3’ |
| *Nrf2* | F:5’-TCACACGAGATGAGCTTAGGGCAA-3’ | R:5’-TACAGTTCTGGGCGGCGACTTTAT-3’ |
| *Acta1* | F:5’- CTTTATCGGTATGGAGTCTGCG-3’ | R:5’- CCGATCCACACTGAGTACTTG -3’ |
| *Fn1* | F:5’- CTTGCACGATGATATGGAGA-3’ | R:5’- AGCTGAACACTGGGTGCTAT -3’ |
| *Ucp3* | F:5’-GGAGTCTCACCTGTTTACTGACAACT-3’ | R:5’-GCACAGAAGCCAGCTCCAA-3’ |
| *Gapdh* | F:5’-GCTTCGGCACATATACTAAAAT-3’ | R:5’-GCAACGGAGACGGCAAACC-3’ |

**Supplementary Table 1.** Primers for qPCR assay.

| **id** | **term** | **category** |
| --- | --- | --- |
| GO:0035235 | ionotropic glutamate receptor signaling pathway | biological process |
| GO:0048168 | regulation of neuronal synaptic plasticity | biological process |
| GO:0051966 | regulation of synaptic transmission, glutamatergic | biological process |
| GO:0007269 | neurotransmitter secretion | biological process |
| GO:0007268 | chemical synaptic transmission | biological process |
| GO:0086010 | membrane depolarization during action potential | biological process |
| GO:0048169 | regulation of long-term neuronal synaptic plasticity | biological process |
| GO:0050806 | positive regulation of synaptic transmission | biological process |
| GO:0007616 | long-term memory | biological process |
| GO:0019228 | neuronal action potential | biological process |
| GO:0042734 | presynaptic membrane | cellular component |
| GO:0043195 | terminal bouton | cellular component |
| GO:0045202 | synapse | cellular component |
| GO:0030424 | axon | cellular component |
| GO:0098839 | postsynaptic density membrane | cellular component |
| GO:0008021 | synaptic vesicle | cellular component |
| GO:0043025 | neuronal cell body | cellular component |
| GO:0043198 | dendritic shaft | cellular component |
| GO:0043005 | neuron projection | cellular component |
| GO:0043229 | intracellular organelle | cellular component |
| GO:1904315 | transmitter-gated ion channel activity involved in regulation of postsynaptic membrane potential | molecular function |
| GO:0017075 | syntaxin-1 binding | molecular function |
| GO:0005234 | extracellularly glutamate-gated ion channel activity | molecular function |
| GO:0004970 | ionotropic glutamate receptor activity | molecular function |
| GO:0005216 | ion channel activity | molecular function |
| GO:0008066 | glutamate receptor activity | molecular function |
| GO:0005261 | cation channel activity | molecular function |
| GO:0000149 | SNARE binding | molecular function |
| GO:0042802 | identical protein binding | molecular function |
| GO:0019905 | syntaxin binding | molecular function |

**Supplementary Table 2.** Top 30 GO term between MI/HKE and MI/CT group.

| **Symbol** | **Impact by HK** | **Description** |
| --- | --- | --- |
| [Bcat1](https://www.genecards.org/cgi-bin/carddisp.pl?gene=BCAT1&keywords=Bcat1) | Downregulated |  |
| [Scg2](https://www.genecards.org/cgi-bin/carddisp.pl?gene=SCG2&keywords=scg2) | Downregulated |  |
| [Col9a2](https://www.genecards.org/cgi-bin/carddisp.pl?gene=COL9A2&keywords=Col9a2) | Downregulated |  |
| Pcdh9 | Downregulated |  |
| Dclk3 | Downregulated |  |
| Ereg | Downregulated | Ereg promotes angiogenesis and inhibits left ventricular remodeling([Cai et al., 2019](#_ENREF_2)) |
| Esm1 | Downregulated |  |
| Ier5l | Downregulated |  |
| Zbtb16 | Downregulated | Zbtb16 induces the spontaneously hypertensive rat to hypertension, left ventricular hypertrophy, and interstitial fibrosis([Liska et al., 2014](#_ENREF_4)) |
| Rad51ap1 | Downregulated |  |
| Cdca7l | Downregulated |  |
| 1810041L15Rik | Downregulated |  |
| Fhad1 | Downregulated | Heart dysfunction biomarkers([di Salvo et al., 2015](#_ENREF_3)) |
| Col2a1 | Downregulated |  |
| Tbx15 | Downregulated |  |
| Trim59 | Downregulated | Trim59 reduces the level of inflammation and apoptosis of myocardial cells caused by I/R([Lv et al., 2020](#_ENREF_5)) |
| Prss12 | Downregulated | Down-regulated in DCM([Wang et al., 2016](#_ENREF_8)) |
| Gdf6 | Downregulated |  |
| Mgat5b | Downregulated |  |
| Nmrk2 | Downregulated | Nmrk2 alleviates ischemia-induced heart failure ([Ahmad et al., 2020](#_ENREF_1)) |
| Fhdc1 | Downregulated |  |
| Sprr1a | Downregulated | SPRR1A protects cardiomyocytes against ischemic injury([Pradervand et al., 2004](#_ENREF_7)) |
| Ano5 | Upregulated |  |
| Retnla | Upregulated |  |
| Zfp947 | Upregulated |  |
| Acot3 | Upregulated |  |
| D7Ertd443e | Upregulated |  |
| Ucp3 | Upregulated | Ucp3 Plays a critical role in cardioprotection against I/R injury and the IPC phenomenon([Ozcan et al., 2013](#_ENREF_6)) |
| Ccl11 | Upregulated |  |
| Pfkfb1 | Upregulated |  |
| C1qtnf4 | Upregulated |  |
| Aldob | Upregulated |  |
| Cyp1a1 | Upregulated |  |

**Supplementary Table 3.** Genes regulated by HKE in post-MI HF.

| **Symbol** | **FPKM(Sham/CT)** | | | **FPKM(MI/CT)** | | | **FPKM(MI/HKE)** | | |
| --- | --- | --- | --- | --- | --- | --- | --- | --- | --- |
| Bcat1 | 0.05 | 0.19 | 0.16 | 0.24 | 0.48 | 0.44 | 0.17 | 0.07 | 0.33 |
| Scg2 | 0.13 | 0.05 | 0 | 1.28 | 0.99 | 1.58 | 0.78 | 0.4 | 0.2 |
| Col9a2 | 0.05 | 0 | 0.11 | 0.6 | 0.42 | 0.85 | 0.13 | 0.15 | 0.12 |
| Pcdh9 | 0.29 | 0.26 | 0.34 | 1.24 | 1.1 | 1.49 | 0.63 | 0.74 | 0.48 |
| Dclk3 | 0.12 | 0.1 | 0.04 | 1.92 | 2.23 | 2.54 | 1.08 | 0.63 | 1.21 |
| Ereg | 0.03 | 0.03 | 0.04 | 0.32 | 0.69 | 0.9 | 0.34 | 0.2 | 0.22 |
| Esm1 | 0.29 | 0.69 | 0.66 | 2.39 | 2.31 | 2 | 0.76 | 1.02 | 0.77 |
| Ier5l | 1.39 | 2.01 | 2.03 | 3.69 | 3.99 | 5.28 | 1.88 | 1.8 | 1.51 |
| Zbtb16 | 5.7 | 7.12 | 7.22 | 14.17 | 23.74 | 21.07 | 5.29 | 9.03 | 7.91 |
| Rad51ap1 | 0.08 | 0.13 | 0 | 0.64 | 0.58 | 0.87 | 0.06 | 0.24 | 0.18 |
| Cdca7l | 0.06 | 0.13 | 0.23 | 0.46 | 1.16 | 0.4 | 0.31 | 0.16 | 0.12 |
| 1810041L15Rik | 0.32 | 0.1 | 0.1 | 0.33 | 0.69 | 0.49 | 0.25 | 0.11 | 0.23 |
| Fhad1 | 0.36 | 0.08 | 0.1 | 0.61 | 0.88 | 0.46 | 0.19 | 0.11 | 0.28 |
| Col2a1 | 0.14 | 0 | 0 | 0.53 | 0.53 | 0.16 | 0.09 | 0.03 | 0.09 |
| Tbx15 | 0.13 | 0.11 | 0.13 | 0.97 | 1.1 | 0.48 | 0.57 | 0.08 | 0.42 |
| Trim59 | 0.35 | 0.24 | 0.31 | 1.72 | 1.57 | 1.26 | 1.07 | 0.43 | 0.7 |
| Prss12 | 0.11 | 0.05 | 0.06 | 0.95 | 1.33 | 0.58 | 0.46 | 0.11 | 0.49 |
| Gdf6 | 0.86 | 0.16 | 0.13 | 2.47 | 2.92 | 2.06 | 1.31 | 0.82 | 1.19 |
| Mgat5b | 0.24 | 0.03 | 0.04 | 0.66 | 1 | 0.55 | 0.49 | 0.15 | 0.3 |
| Nmrk2 | 1 | 10.71 | 2.7 | 10.75 | 22.55 | 20.64 | 12.65 | 4.14 | 6.85 |
| Fhdc1 | 0.12 | 0.12 | 0.12 | 0.38 | 0.46 | 0.4 | 0.27 | 0.05 | 0.15 |
| Sprr1a | 0.23 | 0.74 | 0 | 48.17 | 68.53 | 62.45 | 51.71 | 16.95 | 20.2 |
| Ano5 | 0.29 | 0.22 | 0.15 | 0.17 | 0.06 | 0.05 | 0.33 | 0.14 | 0.27 |
| Retnla | 11.28 | 9.06 | 12.2 | 2.54 | 6.25 | 4.45 | 11.58 | 4.58 | 11.46 |
| Zfp947 | 1.14 | 0.91 | 1.69 | 1.23 | 1.34 | 0.98 | 1.27 | 1.76 | 1.25 |
| Acot3 | 0.83 | 0.93 | 0.95 | 0.34 | 0.27 | 0.49 | 0.85 | 0.98 | 0.45 |
| D7Ertd443e | 1.25 | 1.32 | 1.55 | 1.06 | 1 | 0.46 | 1.33 | 1.43 | 1.32 |
| Ucp3 | 14.43 | 16.91 | 12.42 | 7.42 | 6.97 | 9.19 | 17.9 | 18.66 | 14.08 |
| Ccl11 | 6.4 | 6.28 | 6.2 | 1.59 | 2.85 | 1.31 | 3.98 | 3.27 | 3.54 |
| Pfkfb1 | 9.28 | 8.77 | 9.08 | 2.52 | 2.65 | 2.39 | 6.49 | 6.06 | 2.96 |
| C1qtnf4 | 3.38 | 3.67 | 6.23 | 1.09 | 1.59 | 1.45 | 3.1 | 2.85 | 4.36 |
| Aldob | 4.95 | 6.16 | 7.49 | 2.29 | 0.98 | 1.05 | 2.52 | 4.27 | 5.51 |
| Cyp1a1 | 0.51 | 0.95 | 1.32 | 0.16 | 0.05 | 0.16 | 0.37 | 0.93 | 0.67 |
| Bcat1 | 0.05 | 0.19 | 0.16 | 0.24 | 0.48 | 0.44 | 0.17 | 0.07 | 0.33 |

**Supplementary Table 4.** FPKM values of genes regulated by HKE in post-MI HF.

**LC/MS detail method**

Chromatographic separation was achieved on Waters UPLC BEH C18 1.7µm, 2.1 mm × 50 mm column. The mobile phase A was deionized water. The mobile phase B was methanol. A 2.8 min binary gradient elution (delivered at 0.5 mL/min) was performed for the separation: an isocratic elution of 60 % solvent B for the initial 0.01 min, followed by a linear gradient elution of 60-90% mobile phase B from 0.01 to 1.0 min, and hold the composition of 90% mobile phase B for the next 1.8 min. The mass spectrometry parameters were as follows: negative mode; ion spray voltage, -4500 V; temperature, 500℃; curtain gas, 20 psi; ion source gas1, 60 psi; ion source gas 2, 60 psi; entrance potential, 10 V; delustering potential, -100 and -105 V; collision energy, -45 and -36 eV; and collision cell exit potential, 10 V. The multiple reaction mode transitions were m/z 265.1 → 223.0 for honokiol and m/z 316.2 → 248.1 for internal standard (D3-cannabidiol).

**References:**

Ahmad, F., Tomar, D., Aryal, A.C.S., Elmoselhi, A.B., Thomas, M., Elrod, J.W., et al. (2020). Nicotinamide riboside kinase-2 alleviates ischemia-induced heart failure through P38 signaling. *Biochim Biophys Acta Mol Basis Dis* 1866(3)**,** 165609. doi: 10.1016/j.bbadis.2019.165609.

Cai, Y., Xie, K.L., Wu, H.L., and Wu, K. (2019). Functional suppression of Epiregulin impairs angiogenesis and aggravates left ventricular remodeling by disrupting the extracellular-signal-regulated kinase1/2 signaling pathway in rats after acute myocardial infarction. *J Cell Physiol* 234(10)**,** 18653-18665. doi: 10.1002/jcp.28503.

di Salvo, T.G., Yang, K.C., Brittain, E., Absi, T., Maltais, S., and Hemnes, A. (2015). Right ventricular myocardial biomarkers in human heart failure. *J Card Fail* 21(5)**,** 398-411. doi: 10.1016/j.cardfail.2015.02.005.

Liska, F., Mancini, M., Krupkova, M., Chylikova, B., Krenova, D., Seda, O., et al. (2014). Plzf as a candidate gene predisposing the spontaneously hypertensive rat to hypertension, left ventricular hypertrophy, and interstitial fibrosis. *Am J Hypertens* 27(1)**,** 99-106. doi: 10.1093/ajh/hpt156.

Lv, Z.Q., Yang, C.Y., and Xing, Q.S. (2020). TRIM59 attenuates inflammation and apoptosis caused by myocardial ischemia reperfusion injury by activating the PI3K/Akt signaling pathway. *Eur Rev Med Pharmacol Sci* 24(7)**,** 4005-4015. doi: 10.26355/eurrev_202004_20870.

Ozcan, C., Palmeri, M., Horvath, T.L., Russell, K.S., and Russell, R.R., 3rd (2013). Role of uncoupling protein 3 in ischemia-reperfusion injury, arrhythmias, and preconditioning. *Am J Physiol Heart Circ Physiol* 304(9)**,** H1192-1200. doi: 10.1152/ajpheart.00592.2012.

Pradervand, S., Yasukawa, H., Muller, O.G., Kjekshus, H., Nakamura, T., St Amand, T.R., et al. (2004). Small proline-rich protein 1A is a gp130 pathway- and stress-inducible cardioprotective protein. *EMBO J* 23(22)**,** 4517-4525. doi: 10.1038/sj.emboj.7600454.

Wang, L., Zhu, L., Luan, R., Wang, L., Fu, J., Wang, X., et al. (2016). Analyzing gene expression profiles in dilated cardiomyopathy via bioinformatics methods. *Braz J Med Biol Res* 49(10)**,** e4897. doi: 10.1590/1414-431X20164897.
